# Supplementary material for: Cohort profile: Study on Zika virus infection in Brazil (ZIKABRA study)
Source: PLoS One. 2021 Jan 5;16(1):e0244981. doi: 10.1371/journal.pone.0244981 (PMC7785242; doi:10.1371/journal.pone.0244981)
Supplement: S1 File — (PDF) [file pone.0244981.s001.pdf]

PRE

Número de triagem: ______________________

Repetir Número de triagem:
Repeat Screening number: ______________________

"Número de Triagem" e "Repetir Número de Triagem" estão diferentes, por favor verificar!

"Screening number" and "Repeat screening number" are different, please verify!

Se Centro = 51 (Manaus - FMT - Outpatient ou Manaus - FMT -
Study clinic), então o NÚMERO DE TRIAGEM deve ser entre 40001 - 40999 ou 42001 - 42999!

If Centre = 51 (Manaus - FMT - Outpatient or Manaus - FMT - Study clinic), then SCREENING NUMBER should be between 40001 - 40999 or 42001 - 42999!

Se Centro = 81 (Rio de Janeiro - FIOCRUZ), então o NÚMERO DE TRIAGEM deve ser entre 60001 - 60999!

If Centre = 81 (Rio de Janeiro - FIOCRUZ) then SCREENING NUMBER should be between 60001 - 60999!

Se Centro = 91 (Recife-HC), então o NÚMERO DE TRIAGEM deve ser entre 30001 - 30999 ou 32001 - 32999 ou 34001 - 34999!

If Centre = 91 (Recife-HC), then SCREENING NUMBER should be between 30001 - 30999 or 32001 - 32999 or 34001 - 34999!

Se Centro = 92 (Recife-UPA-Caxangá-Clinic or Recife-UPA-Caxangá-Home), então o NÚMERO DE TRIAGEM deve ser entre 30001 - 30999 ou 32001 - 32999 ou 34001 - 34999!

If Centre = 92 (Recife-UPA-Caxangá-Clinic or Recife-UPA-Caxangá-Home), then SCREENING NUMBER should be between 30001 - 30999 or 32001 - 32999 or 34001 - 34999!

A65921 - Persistência do vírus Zika nos fluidos corporais de pacientes com infecção pelo vírus Zika
Questionário de Pré-triagem

A65921 - Persistence of Zika virus in body fluids of patients with Zika virus infection
Pre-screening Questionnaire

Centro:
Centre:

- ◯ 51 = Manaus-FMT
- ◯ 81 = Rio de Janeiro-FIOCRUZ
- ◯ 91 = Recife-HC
- ◯ 92 = Recife-UPA-Caxangá

INFORMAÇÕES PRE-TRIAGEM

1. a) Data da pre-triagem:<br>1. a) Date of pre-screening:	______________________
b) Profissional de saúde que realizou a pré-triagem (iniciais):<br>b) Pre-screened by (study staff initials):	◯ LHM = Luiz Maciel<br>◯ FAF = Francielen de Azevedo Furtado<br>◯ PCT = Pâmela<br>◯ NMR = Nágila Morais Rocha<br>◯ CAB = Camila Botto
b) Profissional de saúde que realizou a pré-triagem (iniciais):<br>b) Pre-screened by (study staff initials):	◯ FFS = Fernanda Figueiredo<br>◯ KEV = Kennya Valenca
c) Qual e a origem do encaminhamento desta pessoa?<br>c) What is the source of of referal for this person?	◯ 1 = Recife - Contatante (Recife - Contact)<br>◯ 2 = Recife - Posto de Saúde (Recife - Health center)<br>◯ 3 = Recife - Serviço privado (Recife - Private hospital)<br>◯ 99 = Outro (Other)
Se Outro, especificar:<br>If Other, specify:	______________________
2. a) A pessoa é um potencial índice ou um contactante?<br>2. a) Is this person a potential index case or a contact case?	◯ 1 = Potencial índice (Potential index)<br>◯ 2 = Contactante (Contact)
b) Se contactante, número do caso índice:<br>b) If contact, report study ID for index case:	______________________

Se Centro = 51 (Manaus - FMT), então "número do caso índice" deve ser entre 151001 - 151300 ou 251001 - 251300!

If Centre = 51 (Manaus - FMT), then "study ID for index case" should be between 151001 - 151300 or 251001 - 251300!

Se Centro = 81 (Rio de Janeiro - FIOCRUZ), então "número do caso índice" deve ser entre 181001 - 181300 ou 281001 - 281300!

If Centre = 81 (Rio de Janeiro - FIOCRUZ), then "study ID for index case" should be between 181001 - 181300 or 281001 - 281300!

Se Centro = 91 (Recife - HC), então "número do caso índice" deve ser entre 191001 - 191300 ou 291001 - 291300!

If Centre = 91 (Recife - HC), then "study ID for index case" should be between 191001 - 191300 or 291001 - 291300!

Se Centro = 92 (Recife-UPA-Caxangá-Clinic or Recife-UPA-Caxangá-Home), então "número do caso índice" deve ser entre 191001 - 191300 ou 291001 - 291300!

If Centre = 92 (Recife-UPA-Caxangá-Clinic or Recife-UPA-Caxangá-Home), then "study ID for index case" should be between 191001 - 191300 or 291001 - 291300!

c) Se contato, qual o relacionamento com o caso índice?<br>c) If contact, relationship with index case?	◯ 1 = Marido/companheiro (Husband)<br>◯ 2 = Esposa/companheira (Wife)<br>◯ 3 = Parceria sexual (outro) (Sexual partner (other))<br>◯ 4 = Pai (Father)<br>◯ 5 = Mãe (Mother)<br>◯ 6 = Irmão (Brother)<br>◯ 7 = Irmã (Sister)<br>◯ 8 = Filho (Son)<br>◯ 9 = Filha (Daughter)<br>◯ 10 = Avô (Grand father)<br>◯ 11 = Avó (Grand mother)<br>◯ 12 = Outro (Other)
Se Outro, especificar:<br>If Other, specify:	__________________________________
3. a) Esta pessoa já participou de prévia triagem para este estudo?<br>3. a) Has this person already been screened for this study in the past?	◯ 0 = Não (No)<br>◯ 1 = Sim (Yes)
b) Se Sim, preencha o número de triagem inicial:<br>b) If Yes, report initial screening number:	__________________________________

Se Centro = 51 (Manaus - FMT - Outpatient ou Manaus - FMT - Study clinic), então o NÚMERO DE TRIAGEM deve ser entre 40001 - 40999 ou 42001 - 42999!

If Centre = 51 (Manaus - FMT - Outpatient or Manaus - FMT - Study clinic), then SCREENING NUMBER should be between 40001 - 40999 or 42001 - 42999!

Se Centro = 81 (Rio de Janeiro - FIOCRUZ), então o NÚMERO DE TRIAGEM deve ser entre 60001 - 60999!

If Centre = 81 (Rio de Janeiro - FIOCRUZ) then SCREENING NUMBER should be between 60001 - 60999!

Se Centro = 91 (Recife-HC), então o NÚMERO DE TRIAGEM deve ser entre 30001 - 30999 ou 32001 - 32999 ou 34001 - 34999!

If Centre = 91 (Recife-HC), then SCREENING NUMBER should be between 30001 - 30999 or 32001 - 32999 or 34001 - 34999!

Se Centro = 92 (Recife-UPA-Caxangá-Clinic or Recife-UPA-Caxangá-Home), então o NÚMERO DE TRIAGEM deve ser entre 30001 - 30999 ou 32001 - 32999 ou 34001 - 34999!

If Centre = 92 (Recife-UPA-Caxangá-Clinic or Recife-UPA-Caxangá-Home), then SCREENING NUMBER should be between 30001 - 30999 or 32001 - 32999 or 34001 - 34999!

4. Gênero do participante (autoreferido)?<br>4. What is the person gender (Self-referenced)?	◯ 1 = Masculino (Male)<br>◯ 2 = Feminino (Female)<br>◯ 3 = Outro (Other)
Se Outro, especificar:<br>If Other, specify:	__________________________________
5. a) Qual é a idade da pessoa hoje?<br>5. a) What is the person's age today?	__________________________________<br>(Anos)

b) Qual é a data de nascimento da pessoa?
b) What is the person's date of birth? __________________________________

Calculo idade __________________________________

Participante menor de 18 anos, NÃO elegível para o estudo!

Participant under 18, NOT eligible for the study!

Idades diferentes! Por favor verificar.

Different ages! Please verify.

ELIGIBILIDADE PARA O PROCESSO DE CONSENTIMENTO DE TRIAGEM
ELIGIBILITY FOR SCREENING CONSENT PROCESS

Selecione a opção apropriada
Select the appropriate option

Se 5.a) Idade >= 18, então 6.b) deveria ser "Sim"

If 5.a) Age >= 18, then 6.b) should be "Yes"

Se 5.a) Idade < 18, então 6.b) deveria ser "Não"

If 5.a) Age < 18, then 6.b) should be "No"

	Não (No)	Sim (Yes)
6. a) Se a pessoa é um potential índice, a pessoa tem rash?<br>6. a) If the person is a potential index, does the person present a rash?	○	○
6. b) A pessoa tem idade igual ou superior a 18 anos?<br>b) Is the person aged 18 or above?	○	○
6. c) A pessoa vive numa area que permita que ela frequente as visitas do estudo e planeja permanecer nesta residencia durante os próximos 12 meses?<br>c) Does the person live in an area that allows for frequent study visits and plan to stay in this area for the next 12 months?	○	○

6. d) A pessoa é capaz de fornecer o consentimento?
d) Is the person able to provide consent?

○ ○

6. e) Se gênero feminino, a pessoa está grávida?
e) If the person is a woman, is this person pregnant?

○ ○

Participante não elegível para o estudo!

Participant not eligible for study!

---

7. A pessoa é elegível para o processo de consentimento?
7. Is the person eligible for the consent process?

○ 0 = Não (No)
○ 1 = Sim (Yes)

Participante não elegível para o estudo! 7. Deveria ser "Não"

Participant not eligible for study! 7. Should be "No"

CONSENTIMENTO (TRIAGEM)
CONSENT (SCREENING)

8. A pessoa assinou o consentimento para a triagem?
8. Has the person signed the consent for screening?

○ 0 = Não (No)
○ 1 = Sim (Yes)

GÊNERO E TESTE DE GRAVIDEZ
GENDER AND PREGNANCY TEST

9. a) Qual é o gênero atribuído à pessoa no nascimento?
9. a) What is the person assigned gender at birth?

○ 1 = Masculino biológico (Biological male)
○ 2 = Feminino biológico (Biological female)

---

b) 1. Se biológico feminino no nascimento, ela é permanentemente incapaz de engravidar?
b) 1. If the person is biological female at birth, is she permanently unable to have children?

○ 0 = Não (No)
○ 1 = Sim (Yes)

---

2. Se Sim, qual é a razão?
2. If Yes, what is the reason?

○ 1 = Menopausa (Menopause)
○ 2 = Histerectomia (Hysterectomy)
○ 3 = Ligadura tubária (Tubal ligation)
○ 4 = Ovariectomia bilateral (Bilateral ovariectomy)
○ 5 = Outro (Other)

Se Outro, especificar:
If Other, specify:

____

---

c) Se biológico feminino no nascimento e capaz de engravidar, resultado do teste de gravidez?
c) If biological female at birth and able to have children, pregnancy test result?

○ 0 = Negativo (Negative)
○ 1 = Positivo (Positive)
○ 2 = Recusou (Refused)

10. Se biológico feminino no nascimento e capaz de engravidar, teste de gravidez é positivo?<br>10. If biological female at birth and able to have children, is the pregnancy test positive?	◯ 0 = Não (No)<br>◯ 1 = Sim (Yes)
11. a) Se biológico feminino no nascimento, a pessoa está amamentando?<br>11. a) If biological female at birth, is the person lactating?	◯ 0 = Não (No)<br>◯ 1 = Sim (Yes)
b) Se Sim, qual é a data de nascimento da criança?<br>b) If Yes, what is the child's date of birth?	____________________

ELIGIBILIDADE PARA TESTE DE AMOSTRAS
ELIGIBILITY FOR SPECIMENS TESTING

12. A pessoa é elegível para triagem da presença do virus Zika no sangue e na urina (PCR)?<br>12. Is the person eligible for blood and urine Zika virus PCR screening?	◯ 0 = Não (No)<br>◯ 1 = Sim (Yes)

Observações:
Remarks:
