# Supplementary material for: Cohort profile: Study on Zika virus infection in Brazil (ZIKABRA study)
Source: PLoS One. 2021 Jan 5;16(1):e0244981. doi: 10.1371/journal.pone.0244981 (PMC7785242; doi:10.1371/journal.pone.0244981)
Supplement: S2 File — (PDF) [file pone.0244981.s002.pdf]

TRI

Número de triagem: ______________________________

A65921 - Persistência do vírus Zika nos fluidos corporais de pacientes com infecção pelo vírus Zika
Questionário de Triagem

A65921 - Persistence of Zika virus in body fluids of patients with Zika virus infection
Screening Questionnaire

Centro:

- ◯ 51 = Manaus - FMT
- ◯ 81 = Rio de Janeiro - FIOCRUZ
- ◯ 91 = Recife - HC
- ◯ 92 = Recife - UPA - Caxangá

Número de triagem:
Screening number: ______________________________

Repetir Número de triagem:
Repeat Screening number: ______________________________

"Número de Triagem" e "Repetir Número de Triagem" estão diferentes, por favor verificar!

"Screening number" and "Repeat screening number" are different, please verify!

Se Centro = 51 (Manaus - FMT - Outpatient ou Manaus - FMT - Study clinic), então o NÚMERO DE TRIAGEM deve ser entre 40001 - 40999 ou 42001 - 42999!

If Centre = 51 (Manaus - FMT - Outpatient or Manaus - FMT - Study clinic), then SCREENING NUMBER should be between 40001 - 40999 or 42001 - 42999!

Se Centro = 81 (Rio de Janeiro - FIOCRUZ), então o NÚMERO DE TRIAGEM deve ser entre 60001 - 60999!

If Centre = 81 (Rio de Janeiro - FIOCRUZ) then SCREENING NUMBER should be between 60001 - 60999!

Se Centro = 91 (Recife-HC), então o NÚMERO DE TRIAGEM deve ser entre 30001 - 30999 ou 32001 - 32999 ou 34001 - 34999!

If Centre = 91 (Recife-HC), then SCREENING NUMBER should be between 30001 - 30999 or 32001 - 32999 or 34001 - 34999!

Se Centro = 92 (Recife-UPA-Caxangá-Clinic or Recife-UPA-Caxangá-Home), então o NÚMERO DE TRIAGEM deve ser entre 30001 - 30999 ou 32001 - 32999 ou 34001 - 34999!

If Centre = 92 (Recife-UPA-Caxangá-Clinic or Recife-UPA-Caxangá-Home), then SCREENING NUMBER should be between 30001 - 30999 or 32001 - 32999 or 34001 - 34999!

INFORMAÇÕES DE TRIAGEM
SCREENING INFORMATION

1. a) Data da triagem:
1. a) Date of screening: ______________________

b) Profissional de saúde que realizou a triagem (iniciais):
b) Screened by (study staff initials):

- ◯ LHM = Luiz Maciel
- ◯ FAF = Francielen de Azevedo Furtado
- ◯ PCT = Pâmela
- ◯ NMR = Nágila Morais Rocha
- ◯ CAB = Camila Botto

b) Profissional de saúde que realizou a triagem (iniciais):
b) Screened by (study staff initials):

- ◯ FFS = Fernanda Figueiredo
- ◯ KEV = Kennya Valenca

SINTOMAS DE ZIKA
ZIKA SYMPTOMS

2. Temperatura:
2. Temperature: ______________________

(Formato XX.X °CUtilizar ponto ao invés de vírgula para casas decimaisFormat XX.X °C Use period (full stop) instead of comma for decimal places)

3. Presença dos seguintes sintomas:
3. Presence of the following symptoms:

a) Febre? - Nos últimos 30 dias:
a) Fever? - During the last 30 days

Hoje:
Today:

- ◯ 0 = Não (No)
- ◯ 1 = Sim (Yes)

Nos últimos 30 dias:
During the last 30 days:

- ◯ 0 = Não (No)
- ◯ 1 = Sim (Yes)

Se estiver presente, data de início dos síntomas:
If present, date symptom started ______________________

Data maior que 60 dias antes da data da Triagem, favor verificar!
Date greater than 60 days prior to Screening date, please verify!

Se dia desconhecido, por favor preencher "Mês" e "Ano" abaixo!
If day is unknown, please enter "Month" and "Year" below!

Mês:<br>Month:	◯ Desconhecido<br>◯ Jan<br>◯ Fev<br>◯ Mar<br>◯ Abr<br>◯ Mai<br>◯ Jun<br>◯ Jul<br>◯ Ago<br>◯ Set<br>◯ Out<br>◯ Nov<br>◯ Dez

Ano:<br>Year:	◯ Desconhecido<br>◯ 1970<br>◯ 1971<br>◯ 1972<br>◯ 1973<br>◯ 1974<br>◯ 1975<br>◯ 1976<br>◯ 1977<br>◯ 1978<br>◯ 1979<br>◯ 1980<br>◯ 1981<br>◯ 1982<br>◯ 1983<br>◯ 1984<br>◯ 1985<br>◯ 1986<br>◯ 1987<br>◯ 1988<br>◯ 1989<br>◯ 1990<br>◯ 1991<br>◯ 1992<br>◯ 1993<br>◯ 1994<br>◯ 1995<br>◯ 1996<br>◯ 1997<br>◯ 1998<br>◯ 1999<br>◯ 2000<br>◯ 2001<br>◯ 2002<br>◯ 2003<br>◯ 2004<br>◯ 2005<br>◯ 2006<br>◯ 2007<br>◯ 2008<br>◯ 2009<br>◯ 2010<br>◯ 2011<br>◯ 2012<br>◯ 2013<br>◯ 2014<br>◯ 2015<br>◯ 2016<br>◯ 2017<br>◯ 2018<br>◯ 2019
b) 1. Erupção cutânea?<br>b) 1. Skin rash?	
Hoje:<br>Today:	◯ 0 = Não (No)<br>◯ 1 = Sim (Yes)
Nos últimos 30 dias:<br>During the last 30 days	◯ 0 = Não (No)<br>◯ 1 = Sim (Yes)

Se estiver presente, data de início dos síntomas:
If present, date symptom started ________________________________

Data maior que 60 dias antes da data da Triagem, favor verificar!
Date greater than 60 days prior to Screening date, please verify!

Se dia desconhecido, por favor preencher "Mês" e "Ano" abaixo!
If day is unknown, please enter "Month" and "Year" below!

Mês:
Month:

- ◯ Desconhecido
- ◯ Jan
- ◯ Fev
- ◯ Mar
- ◯ Abr
- ◯ Mai
- ◯ Jun
- ◯ Jul
- ◯ Ago
- ◯ Set
- ◯ Out
- ◯ Nov
- ◯ Dez

Ano:<br>Year:	◯ Desconhecido<br>◯ 1970<br>◯ 1971<br>◯ 1972<br>◯ 1973<br>◯ 1974<br>◯ 1975<br>◯ 1976<br>◯ 1977<br>◯ 1978<br>◯ 1979<br>◯ 1980<br>◯ 1981<br>◯ 1982<br>◯ 1983<br>◯ 1984<br>◯ 1985<br>◯ 1986<br>◯ 1987<br>◯ 1988<br>◯ 1989<br>◯ 1990<br>◯ 1991<br>◯ 1992<br>◯ 1993<br>◯ 1994<br>◯ 1995<br>◯ 1996<br>◯ 1997<br>◯ 1998<br>◯ 1999<br>◯ 2000<br>◯ 2001<br>◯ 2002<br>◯ 2003<br>◯ 2004<br>◯ 2005<br>◯ 2006<br>◯ 2007<br>◯ 2008<br>◯ 2009<br>◯ 2010<br>◯ 2011<br>◯ 2012<br>◯ 2013<br>◯ 2014<br>◯ 2015<br>◯ 2016<br>◯ 2017<br>◯ 2018<br>◯ 2019
2. Erupção máculo-papular?<br>2. Maculopapular rash?	
Hoje:<br>Today:	◯ 0 = Não (No)<br>◯ 1 = Sim (Yes)
Nos últimos 30 dias:<br>During the last 30 days	◯ 0 = Não (No)<br>◯ 1 = Sim (Yes)
c) Coceira na pele (prurido)?<br>c) Skin itching (pruritus)?	

Hoje:<br>Today:	◯ 0 = Não (No)<br>◯ 1 = Sim (Yes)
Nos últimos 30 dias:<br>During the last 30 days	◯ 0 = Não (No)<br>◯ 1 = Sim (Yes)
Se estiver presente, data de início dos síntomas:<br>If present, date symptom started	__________________________________
Data maior que 60 dias antes da data da Triagem, favor verificar!<br>Date greater than 60 days prior to Screening date, please verify!	
Se dia desconhecido, por favor preencher "Mês" e "Ano" abaixo!<br>If day is unknown, please enter "Month" and "Year" below!	
Mês:<br>Month:	◯ Desconhecido<br>◯ Jan<br>◯ Fev<br>◯ Mar<br>◯ Abr<br>◯ Mai<br>◯ Jun<br>◯ Jul<br>◯ Ago<br>◯ Set<br>◯ Out<br>◯ Nov<br>◯ Dez

Ano:
Year:

◯ Desconhecido
◯ 1970
◯ 1971
◯ 1972
◯ 1973
◯ 1974
◯ 1975
◯ 1976
◯ 1977
◯ 1978
◯ 1979
◯ 1980
◯ 1981
◯ 1982
◯ 1983
◯ 1984
◯ 1985
◯ 1986
◯ 1987
◯ 1988
◯ 1989
◯ 1990
◯ 1991
◯ 1992
◯ 1993
◯ 1994
◯ 1995
◯ 1996
◯ 1997
◯ 1998
◯ 1999
◯ 2000
◯ 2001
◯ 2002
◯ 2003
◯ 2004
◯ 2005
◯ 2006
◯ 2007
◯ 2008
◯ 2009
◯ 2010
◯ 2011
◯ 2012
◯ 2013
◯ 2014
◯ 2015
◯ 2016
◯ 2017
◯ 2018
◯ 2019

d) Hiperemia conjuntival sem secreção e sem coceira?
d) Conjunctival hyperemia without secretions and without pruritus?

Hoje:
Today:

◯ 0 = Não (No)
◯ 1 = Sim (Yes)

Nos últimos 30 dias:
During the last 30 days

◯ 0 = Não (No)
◯ 1 = Sim (Yes)

Se estiver presente, data de início dos síntomas:
If present, date symptom started ________________________

Data maior que 60 dias antes da data da Triagem, favor verificar!
Date greater than 60 days prior to Screening date, please verify!

Se dia desconhecido, por favor preencher "Mês" e "Ano" abaixo!
If day is unknown, please enter "Month" and "Year" below!

Mês:
Month:

- ◯ Desconhecido
- ◯ Jan
- ◯ Fev
- ◯ Mar
- ◯ Abr
- ◯ Mai
- ◯ Jun
- ◯ Jul
- ◯ Ago
- ◯ Set
- ◯ Out
- ◯ Nov
- ◯ Dez

Ano:
Year:

◯ Desconhecido
◯ 1970
◯ 1971
◯ 1972
◯ 1973
◯ 1974
◯ 1975
◯ 1976
◯ 1977
◯ 1978
◯ 1979
◯ 1980
◯ 1981
◯ 1982
◯ 1983
◯ 1984
◯ 1985
◯ 1986
◯ 1987
◯ 1988
◯ 1989
◯ 1990
◯ 1991
◯ 1992
◯ 1993
◯ 1994
◯ 1995
◯ 1996
◯ 1997
◯ 1998
◯ 1999
◯ 2000
◯ 2001
◯ 2002
◯ 2003
◯ 2004
◯ 2005
◯ 2006
◯ 2007
◯ 2008
◯ 2009
◯ 2010
◯ 2011
◯ 2012
◯ 2013
◯ 2014
◯ 2015
◯ 2016
◯ 2017
◯ 2018
◯ 2019

e) Dor nas articulações (artralgia)?
e) Pain in the joints (arthralgia)?

Hoje:<br>Today:	◯ 0 = Não (No)<br>◯ 1 = Sim, apenas em uma articulação (Yes, only one joint)<br>◯ 2 = Sim, em duas ou mais articulações (Yes, in two joints or more)<br>◯ 3 = Sim, sem informação sobre o número de articulações (Yes, without information on number of joints)
Nos últimos 30 dias:<br>During the last 30 days	◯ 0 = Não (No)<br>◯ 1 = Sim, apenas em uma articulação (Yes, only one joint)<br>◯ 2 = Sim, em duas ou mais articulações (Yes, in two joints or more)<br>◯ 3 = Sim, sem informação sobre o número de articulações (Yes, without information on number of joints)
Se estiver presente, data de início dos síntomas:<br>If present, date symptom started	______________________________
Data maior que 60 dias antes da data da Triagem, favor verificar!<br>Date greater than 60 days prior to Screening date, please verify!	
Se dia desconhecido, por favor preencher "Mês" e "Ano" abaixo!<br>If day is unknown, please enter "Month" and "Year" below!	
Mês:<br>Month:	◯ Desconhecido<br>◯ Jan<br>◯ Fev<br>◯ Mar<br>◯ Abr<br>◯ Mai<br>◯ Jun<br>◯ Jul<br>◯ Ago<br>◯ Set<br>◯ Out<br>◯ Nov<br>◯ Dez

Ano:<br>Year:	◯ Desconhecido<br>◯ 1970<br>◯ 1971<br>◯ 1972<br>◯ 1973<br>◯ 1974<br>◯ 1975<br>◯ 1976<br>◯ 1977<br>◯ 1978<br>◯ 1979<br>◯ 1980<br>◯ 1981<br>◯ 1982<br>◯ 1983<br>◯ 1984<br>◯ 1985<br>◯ 1986<br>◯ 1987<br>◯ 1988<br>◯ 1989<br>◯ 1990<br>◯ 1991<br>◯ 1992<br>◯ 1993<br>◯ 1994<br>◯ 1995<br>◯ 1996<br>◯ 1997<br>◯ 1998<br>◯ 1999<br>◯ 2000<br>◯ 2001<br>◯ 2002<br>◯ 2003<br>◯ 2004<br>◯ 2005<br>◯ 2006<br>◯ 2007<br>◯ 2008<br>◯ 2009<br>◯ 2010<br>◯ 2011<br>◯ 2012<br>◯ 2013<br>◯ 2014<br>◯ 2015<br>◯ 2016<br>◯ 2017<br>◯ 2018<br>◯ 2019
f) Edema periarticular?<br>f) Periarticular edema?	
Hoje:<br>Today:	◯ 0 = Não (No)<br>◯ 1 = Sim (Yes)
Nos últimos 30 dias:<br>During the last 30 days	◯ 0 = Não (No)<br>◯ 1 = Sim (Yes)

Se estiver presente, data de início dos síntomas:
If present, date symptom started ________________________________

Data maior que 60 dias antes da data da Triagem, favor verificar!
Date greater than 60 days prior to Screening date, please verify!

Se dia desconhecido, por favor preencher "Mês" e "Ano" abaixo!
If day is unknown, please enter "Month" and "Year" below!

Mês:
Month:

- ◯ Desconhecido
- ◯ Jan
- ◯ Fev
- ◯ Mar
- ◯ Abr
- ◯ Mai
- ◯ Jun
- ◯ Jul
- ◯ Ago
- ◯ Set
- ◯ Out
- ◯ Nov
- ◯ Dez

Ano:<br>Year:	◯ Desconhecido<br>◯ 1970<br>◯ 1971<br>◯ 1972<br>◯ 1973<br>◯ 1974<br>◯ 1975<br>◯ 1976<br>◯ 1977<br>◯ 1978<br>◯ 1979<br>◯ 1980<br>◯ 1981<br>◯ 1982<br>◯ 1983<br>◯ 1984<br>◯ 1985<br>◯ 1986<br>◯ 1987<br>◯ 1988<br>◯ 1989<br>◯ 1990<br>◯ 1991<br>◯ 1992<br>◯ 1993<br>◯ 1994<br>◯ 1995<br>◯ 1996<br>◯ 1997<br>◯ 1998<br>◯ 1999<br>◯ 2000<br>◯ 2001<br>◯ 2002<br>◯ 2003<br>◯ 2004<br>◯ 2005<br>◯ 2006<br>◯ 2007<br>◯ 2008<br>◯ 2009<br>◯ 2010<br>◯ 2011<br>◯ 2012<br>◯ 2013<br>◯ 2014<br>◯ 2015<br>◯ 2016<br>◯ 2017<br>◯ 2018<br>◯ 2019

Observações:
Remarks:
