# Supplementary material for: Cohort profile: Study on Zika virus infection in Brazil (ZIKABRA study)
Source: PLoS One. 2021 Jan 5;16(1):e0244981. doi: 10.1371/journal.pone.0244981 (PMC7785242; doi:10.1371/journal.pone.0244981)
Supplement: S3 File — (PDF) [file pone.0244981.s003.pdf]

IDE

Número de triagem: ______________________

A65921 - Persistência do vírus Zika nos fluidos corporais de pacientes com infecção pelo vírus Zika
Identificação (confidencial)

A65921 - Persistence of Zika virus in body fluids of patients with Zika virus infection
Identification (confidential)

Centro:
Centre:

◯ 51 = Manaus - FMT
◯ 81 = Rio de Janeiro - FIOCRUZ
◯ 91 = Recife - HC
◯ 92 = Recife - UPA - Caxangá

Data da triagem:
Date of screening: ______________________

Número de triagem:
Screening number: ______________________

Repetir Número de triagem:
Repeat Screening number: ______________________

"Número de Triagem" e "Repetir Número de Triagem" estão diferentes, por favor verificar!

"Screening number" and "Repeat screening number" are different, please verify!

Se Centro = 51 (Manaus - FMT - Outpatient ou Manaus - FMT - Study clinic), então o NÚMERO DE TRIAGEM deve ser entre 40001 - 40999 ou 42001 - 42999!

If Centre = 51 (Manaus - FMT - Outpatient or Manaus - FMT - Study clinic), then SCREENING NUMBER should be between 40001 - 40999 or 42001 - 42999!

Se Centro = 81 (Rio de Janeiro - FIOCRUZ), então o NÚMERO DE TRIAGEM deve ser entre 60001 - 60999!

If Centre = 81 (Rio de Janeiro - FIOCRUZ) then SCREENING NUMBER should be between 60001 - 60999!

Se Centro = 91 (Recife-HC), então o NÚMERO DE TRIAGEM deve ser entre 30001 - 30999 ou 32001 - 32999 ou 34001 - 34999!

If Centre = 91 (Recife-HC), then SCREENING NUMBER should be between 30001 - 30999 or 32001 - 32999 or 34001 - 34999!

Se Centro = 92 (Recife-UPA-Caxangá-Clinic or Recife-UPA-Caxangá-Home), então o NÚMERO DE TRIAGEM deve ser entre 30001 - 30999 ou 32001 - 32999 ou 34001 - 34999!

If Centre = 92 (Recife-UPA-Caxangá-Clinic or Recife-UPA-Caxangá-Home), then SCREENING NUMBER should be between 30001 - 30999 or 32001 - 32999 or 34001 - 34999!

Número do CNS:
CNS number: __________________________________

Repetir Número do CNS:
Repeat CNS number: __________________________________

"Número do CNS" e "Repetir Número do CNS" estão diferentes, por favor verificar!

"CNS Number" and "Repeat CNS Number" are different, please verify!

O participante foi recrutado?
Was the participant recruited?
- ◯ 0 = Não (No)
- ◯ 1 = Sim (Yes)

Data do recrutamento:
Recruitment date: __________________________________

Número único de identificação:
Unique ID number: __________________________________

Repetir Número único de identificação:
Repeat Unique ID number: __________________________________

"Número único de identificação" e "Repetir Número único de identificação" estão diferentes, por favor verificar!

"Unique ID number" and "Repeat Unique ID number" are different, please verify!

Se Centro = 51 (Manaus - FMT), então "Número de Identificação Única" deve ser entre 151001 - 151300 ou 251001 - 251300!

If Centre = 51 (Manaus - FMT), then "Unique ID number" should be between 151001 - 151300 or 251001 - 251300!

Se Centro = 81 (Manaus - FMT), então "Número de Identificação Única" deve ser entre 181001 - 181300 ou 281001 - 281300!

If Centre = 81 (Manaus - FMT), then "Unique ID number" should be between 181001 - 181300 or 281001 - 281300!

Se Centro = 91 (Manaus - FMT), então "Número de Identificação Única" deve ser entre 191001 - 191300 ou 291001 - 291300!

If Centre = 91 (Manaus - FMT), then "Unique ID number" should be between 191001 - 191300 or 291001 - 291300!
