# Supplementary material for: Cohort profile: Study on Zika virus infection in Brazil (ZIKABRA study)
Source: PLoS One. 2021 Jan 5;16(1):e0244981. doi: 10.1371/journal.pone.0244981 (PMC7785242; doi:10.1371/journal.pone.0244981)
Supplement: S4 File — (PDF) [file pone.0244981.s004.pdf]

REC

Número de triagem: ________________

A65921 - Persistência do vírus Zika nos fluidos corporais de pacientes com infecção pelo vírus Zika
Questionário de Recrutamento

A65921 - Persistence of Zika virus in body fluids of patients with Zika virus infection
Recruitment Questionnaire

Centro:
Centre:

- ◯ 51 = Manaus - FMT
- ◯ 81 = Rio de Janeiro - FIOCRUZ
- ◯ 91 = Recife - HC

Número de triagem:
Screening number: ________________

Repetir Número de triagem:
Repeat Screening number: ________________

"Número de triagem" e "Repetir Número de triagem" estão diferentes, por favor verificar!

"Screening number" and "Repeat screening number" are different, please verify!

Se Centro = 51 (Manaus - FMT - Outpatient ou Manaus - FMT - Study clinic), então o NÚMERO DE TRIAGEM deve ser entre 40001 - 40999 ou 42001 - 42999!

If Centre = 51 (Manaus - FMT - Outpatient or Manaus - FMT - Study clinic), then SCREENING NUMBER should be between 40001 - 40999 or 42001 - 42999!

Se Centro = 81 (Rio de Janeiro - FIOCRUZ), então o NÚMERO DE TRIAGEM deve ser entre 60001 - 60999!

If Centre = 81 (Rio de Janeiro - FIOCRUZ) then SCREENING NUMBER should be between 60001 - 60999!

Se Centro = 91 (Recife-HC), então o NÚMERO DE TRIAGEM deve ser entre 30001 - 30999 ou 32001 - 32999 ou 34001 - 34999!

If Centre = 91 (Recife-HC), then SCREENING NUMBER should be between 30001 - 30999 or 32001 - 32999 or 34001 - 34999!

ELIGIBILIDADE PARA RECRUTAMENTO
ELIGIBILITY FOR ENROLLMENT

1. a) Data da visita:
1. a) Date of visit: ______________________

b) Profissional de saúde que realizou o recrutamento (iniciais):
b) Enrolled by (study staff initials):
- ◯ LHM = Luiz Maciel
- ◯ FAF = Francielen de Azevedo Furtado
- ◯ PCT = Pâmela
- ◯ NMR = Nágila Morais Rocha
- ◯ CAB = Camila Botto

b) Profissional de saúde que realizou o recrutamento (iniciais):
b) Enrolled by (study staff initials):
- ◯ FFS = Fernanda Figueiredo
- ◯ KEV = Kennya Valenca

2. a) Resultado da triagem - Presença do vírus Zika - Sangue
2. a) Result of Zika virus PCR screening - Blood
- ◯ 0 = Negativo (Negative)
- ◯ 1 = Positivo (Positive)
- ◯ 2 = Inconclusivo (Inconclusive)
- ◯ 3 = Não disponível (Not available)

b) Resultado da triagem - Presença do vírus Zika - Urina
b) Result of Zika virus PCR screening - Urine
- ◯ 0 = Negativo (Negative)
- ◯ 1 = Positivo (Positive)
- ◯ 2 = Inconclusivo (Inconclusive)
- ◯ 3 = Não disponível (Not available)

Selecione a opção apropriada
Select the appropriate option

	Não (No)	Sim (Yes)
3. a) A pessoa assinou o consentimento para o recrutamento?<br>3. a) Has the person signed the consent for enrollment?	◯	◯
b) Resultado positivo para o virus Zika no sangue e/ou na urina?<br>b) Is Zika virus PCR screening result positive in blood and/or urine?	◯	◯

Participante não elegível para o estudo!

Participant not eligible for study!

Se pergunta 3.b) = "Não", entao pergunta 2.a) e b) NÃO deveriam ser "Positivo"!

If question 3.b) = "No", then questions 2.a) and b) should NOT be "Positive"!

Se pergunta 3.b) = "Sim", entao pergunta 2.a) ou b) deveria ser "Positivo"!

If question 3.b) = "Yes", then question 2.a) or b) should be "Positive"!

4. A pessoa é elegível para recrutamento?
4. Is the person eligible for enrollment?

◯ 0 = Não (No)
◯ 1 = Sim (Yes)

5. a) Qual é o gênero atribuído à pessoa no nascimento?
5. a) What is the person's assigned gender at birth?

◯ 1 = Biológico masculino (Biological male)
◯ 2 = Biológico feminino (Biological female)

b) Número único de identificação:
b) Participant's ID number: __________________________________

b) Repetir Número único de identificação:
b) Repeat Participant's ID number: __________________________________

"Número único de identificação" e "Repetir Número único de identificação" estão diferentes, por favor verificar!

"Participant's ID number" and "Repeat Participant's ID number" are different, please verify!

Se Centro = 51 (Manaus - FMT) e "5. a) Qual é o gênero atribuído à pessoa no nascimento? = Biológico masculino", então "b) Identificação Única do Participante" deve ser entre 151001 - 151300!

If Centre = 51 (Manaus - FMT) and "5. a) What is the person's assigned gender at birth? = Biological male", then "b) Participant's ID number" should be between 151001 - 151300!

Se Centro = 51 (Manaus - FMT) e "5. a) Qual é o gênero atribuído à pessoa no nascimento? = Biológico feminino", então "b) Identificação Única do Participante" deve ser entre 251001 - 251300!

If Centre = 51 (Manaus - FMT) and "5. a) What is the person's assigned gender at birth? = Biological female", then "b) Participant's ID number" should be between 251001 - 251300!

Se Centro = 81 (Rio de Janeiro-FIOCRUZ) e "5. a) Qual é o gênero atribuído à pessoa no nascimento? = Biológico masculino", então "b) Identificação Única do Participante" deve ser entre 181001 - 181300!

If Centre = 81 (Rio de Janeiro-FIOCRUZ) and "5. a) What is the person's assigned gender at birth? = Biological male", then "b) Participant's ID number" should be between 181001 - 181300!

Se Centro = 81 (Rio de Janeiro-FIOCRUZ) e "5. a) Qual é o gênero atribuído à pessoa no nascimento? = Biológico feminino", então "b) Identificação Única do Participante" deve ser entre 281001 - 281300!

If Centre = 81 (Rio de Janeiro-FIOCRUZ) and "5. a) What is the person's assigned gender at birth? = Biological female", then "b) Participant's ID number" should be between 281001 - 281300!

Se Centro = 91 (Recife-HC) e "5. a) Qual é o gênero atribuído à pessoa no nascimento? = Biológico masculino", então "b) Identificação Única do Participante" deve ser entre 191001 - 191300!

If Centre = 91 (Recife-HC) and "5. a) What is the person's assigned gender at birth? = Biological male", then "b) Participant's ID number" should be between 191001 - 191300!

Se Centro = 91 (Recife-HC) e "5. a) Qual é o gênero atribuído à pessoa no nascimento? = Biológico feminino", então "b) Identificação Única do Participante" deve ser entre 291001 - 291300!

If Centre = 91 (Recife-HC) and "5. a) What is the person's assigned gender at birth? = Biological female", then "b) Participant's ID number" should be between 291001 - 291300!

c) A pessoa é índice ou contactante?
c) Is this person an index case or a contact case?

◯ 1 = Índice (Index)
◯ 2 = Contactante (Contact)

d) Se conctatante, número único de identificação do caso índice:<br>d) If contact, report study ID for index case:	__________________________________
d) Repetir número único de identificação do caso índice:<br>d) Repeat study ID for index case:	__________________________________
e) Relacionamento com o caso índice?<br>e) If contact, relationship with index case?	◯ 1 = Marido/companheiro (Husband)<br>◯ 2 = Esposa/companheira (Wife)<br>◯ 3 = Parceria sexual (outro) (Sexual partner (other))<br>◯ 4 = Pai (Father)<br>◯ 5 = Mãe (Mother)<br>◯ 6 = Irmão (Brother)<br>◯ 7 = Irmã (Sister)<br>◯ 8 = Filho (Son)<br>◯ 9 = Filha (Daughter)<br>◯ 10 = Avô (Grand father)<br>◯ 11 = Avó (Grand mother)<br>◯ 12 = Outro (Other)
Se Outro, especificar:<br>If Other, specify:	__________________________________

"Número único de identificação" e "Repetir Número único de identificação" estão diferentes, por favor verificar!

"Participant's ID number" and "Repeat Participant's ID number" are different, please verify!

Se Centro = 51 (Manaus - FMT), então "número do caso índice" deve ser entre 151001 - 151300 ou 251001 - 251300!

If Centre = 51 (Manaus - FMT), then "study ID for index case" should be between 151001 - 151300 or 251001 - 251300!

Se Centro = 81 (Manaus - FMT), então "número do caso índice" deve ser entre 181001 - 181300 ou 281001 - 281300!

If Centre = 81 (Manaus - FMT), then "study ID for index case" should be between 181001 - 181300 or 281001 - 281300!

Se Centro = 91 (Manaus - FMT), então "número do caso índice" deve ser entre 191001 - 191300 ou 291001 - 291300!

If Centre = 91 (Manaus - FMT), then "study ID for index case" should be between 191001 - 191300 or 291001 - 291300!

6. a) Consentimento dado para o armazenamento a longo prazo das amostras?<br>6. a) Consent provided for long-term storage of samples?	◯ 0 = Não (No)<br>◯ 1 = Sim (Yes)
b) Se índice, consentimento dado para contactar contactantes domiciliares/parceiro sexual para triagem?<br>b) If index, consent provided for contacting household members/sexual partner for screening?	◯ 0 = Não (No)<br>◯ 1 = Sim (yes)<br>◯ 2 = Vive sozinho e não tem parceiro sexual (Lives alone and has no sexual partner)

ENTREVISTA
INTERVIEW

7. Você classificaria a sua cor de pele/raça como?
7. How do you define your race/skin colour?

- ◯ 1 = Branca (White)
- ◯ 2 = Preta (Black)
- ◯ 3 = Parda (Brown)
- ◯ 4 = Indígena (Indigenous)
- ◯ 5 = Outra (Other)
- ◯ 8 = Não sabe (Don't know)
- ◯ 9 = Recusou (Refused)

Se Outra, especificar:
If Other, specify: ________________________________

8. Qual a sua ocupação?
8. What is your occupation? ________________________________

9. a) Qual a sua escolaridade?
9. a) What is your level of education?

- ◯ 1 = Sem escolaridade (No school)
- ◯ 2 = 1° grau/ensino fundamental completo (Primary complete)
- ◯ 3 = 1° grau/ensino fundamental incompleto (Primary incomplete)
- ◯ 4 = 2° grau/ensino médio completo (Secondary complete)
- ◯ 5 = 2° grau/ensino médio incompleto (Secondary incomplete)
- ◯ 6 = Terceiro grau (University)
- ◯ 7 = Pós-graduação (Post graduated)
- ◯ 8 = Não sabe (Don't know)
- ◯ 9 = Recusou (Refused)

b) Quantos anos de estudo você concluiu?
b) How many years of study did you conclude? ________________________________

10. Qual é o seu estado civil?
10. What is your current relationship status?

- ◯ 1 = Solteiro(a) (Single)
- ◯ 2 = Casado(a) (Married)
- ◯ 3 = União estável (Long-term relationship)
- ◯ 4 = Separado(a) ou divorciado(a) (Separated or divorced)
- ◯ 5 = Viúvo(a) (Widowed)
- ◯ 6 = Outro (Other)
- ◯ 8 = Não sabe (Don't know)
- ◯ 9 = Recusou (Refused)

Se Outro, especificar:
If Other, specify: ________________________________

11. a) Você está vivendo sozinho ou com outras pessoas?
11. a) Are you living alone or with other people?

- ◯ 1 = Sozinho (Alone)
- ◯ 2 = Com outras pessoas (With other people)
- ◯ 9 = Recusou (Refused)

b) Quantas pessoas vivem em seu domicílio, excluindo você?
b) How many people live in your house, excluding yourself?

____
(99 = Recusou99 = Refused)

c) Quantas dessas pessoas têm pelo menos 18 anos de idade?<br>c) How many of those people are at least 18 years old?	______________________________<br>(88 = Não sabe, 99 = Recusou88 = Don't know, 99 = Refused)
12. Peso:<br>12. Weight:	
	______________________________<br>(Kg Formato XXX.XUtilizar ponto ao invés de vírgula para casas decimaisKg Format XXX.X Use period (full stop) instead of comma for decimal places)
	☐ Não medido (Not measured)
13. Altura:<br>13. Height:	
	______________________________<br>(m Formato X.XXUtilizar ponto ao invés de vírgula para casas decimaism Format X.XX Use period (full stop) instead of comma for decimal places)
	☐ Não medida (Not measured)
14. Temperatura:<br>14. Temperature:	
	______________________________<br>(°C Formato XX.XUtilizar ponto ao invés de vírgula para casas decimais°C Format XX.X Use period (full stop) instead of comma for decimal places)
	☐ Não medida (Not measured)
15. Frequência cardíaca:<br>15. Heart rate:	______________________________<br>(Batidas/minBeats/min)
16. Frequência respiratória:<br>16. Respiratory rate:	______________________________<br>(Ciclos/minCycles/min)
17. Pressão arterial (sentado por pelo menos 5 minutos)<br>17. Blood pressure (sitting for at least 5 minutes)	
a) Sistólica:<br>a) Systolic	

____
(mmHg)

☐ Não medida (Not measured)

b) Diastólica:
b) Diastolic

____
(mmHg)

☐ Não medida (Not measured)

Observações:
Remarks:
