# Supplementary material for: Cohort profile: Study on Zika virus infection in Brazil (ZIKABRA study)
Source: PLoS One. 2021 Jan 5;16(1):e0244981. doi: 10.1371/journal.pone.0244981 (PMC7785242; doi:10.1371/journal.pone.0244981)
Supplement: S5 File — (PDF) [file pone.0244981.s005.pdf]

AMO

Número de triagem: ______________________

A65921 - Persistência do vírus Zika nos fluidos corporais de pacientes com infecção pelo vírus Zika
Questionário de Coleta de Amostras

A65921 - Persistence of Zika virus in body fluids of patients with Zika virus infection
Sample Collection Questionnaire

Centro:
Centre:

◯ 51 = Manaus - FMT
◯ 81 = Rio de Janeiro - FIOCRUZ
◯ 91 = Recife - HC

Número único de identificação:
Unique ID number: ______________________

Repetir Número único de identificação:
Repeat Unique ID number: ______________________

"Número único de identificação" e "Repetir Número único de identificação" estão diferentes, por favor verificar!

"Unique ID number" and "Repeat Unique ID number" are different, please verify!

Se Centro = 51 (Manaus - FMT), então "Número de Identificação Única" deve ser entre 151001 - 151300 ou 251001 - 251300!

If Centre = 51 (Manaus - FMT), then "Unique ID number" should be between 151001 - 151300 or 251001 - 251300!

Se Centro = 81 (Manaus - FMT), então "Número de Identificação Única" deve ser entre 181001 - 181300 ou 281001 - 281300!

If Centre = 81 (Manaus - FMT), then "Unique ID number" should be between 181001 - 181300 or 281001 - 281300!

Se Centro = 91 (Manaus - FMT), então "Número de Identificação Única" deve ser entre 191001 - 191300 ou 291001 - 291300!

If Centre = 91 (Manaus - FMT), then "Unique ID number" should be between 191001 - 191300 or 291001 - 291300!

VISITA
VISIT

1. a) Data da visita:
2. a) Date of visit: ______________________

2. a) Número da visita:
1. a) Visit number: ______________________

b) Tipo de visita:
b) Visit type:

◯ 1 = Programada (Scheduled)
◯ 2 = Não programada (Unscheduled)

c) Profissional de saúde que preencheu o questionário (iniciais):<br>c) Health professional who completed the questionnaire (Initials):	◯ LHM = Luiz Maciel<br>◯ FAF = Francielen de Azevedo Furtado<br>◯ PCT = Pâmela<br>◯ NMR = Nágila Morais Rocha<br>◯ CAB = Camila Botto
c) Profissional de saúde que preencheu o questionário (iniciais):<br>c) Health professional who completed the questionnaire (Initials):	◯ FFS = Fernanda Figueiredo<br>◯ KEV = Kennya Valenca

AMOSTRAS
SAMPLES

3. Amostras:<br>3. Samples	
a) Sangue (1)<br>a) Blood (1)	
1. Coleta programada?<br>1. Planned for collection?	◯ 0 = Não (No)<br>◯ 1 = Sim (Yes)
2. Se programada, amostra coletada?<br>2. If due, sample collected?	◯ 0 = Não (No)<br>◯ 1 = Sim (Yes)
3. Se não, por quê?<br>3. If not collected, why?	◯ 1 = Recusou (Refused)<br>◯ 2 = Não conseguiu fornecer (Did not manage to provide)<br>◯ 3 = Outro (Other)
Se Outro, especificar:<br>If Other, specify:	______________________
b) Urina (2)<br>b) Urine (2)	
1. Coleta programada?<br>1. Planned for collection?	◯ 0 = Não (No)<br>◯ 1 = Sim (Yes)
2. Se programada, amostra coletada?<br>2. If due, sample collected?	◯ 0 = Não (No)<br>◯ 1 = Sim (Yes)
3. Se não, por quê?<br>3. If not collected, why?	◯ 1 = Recusou (Refused)<br>◯ 2 = Não conseguiu fornecer (Did not manage to provide)<br>◯ 3 = Outro (Other)
Se Outro, especificar:<br>If Other, specify:	______________________
c) Saliva (3)<br>c) Saliva (3)	
1. Coleta programada?<br>1. Planned for collection?	◯ 0 = Não (No)<br>◯ 1 = Sim (Yes)

2. Se programada, amostra coletada?<br>2. If due, sample collected?	◯ 0 = Não (No)<br>◯ 1 = Sim (Yes)
3. Se não, por quê?<br>3. If not collected, why?	◯ 1 = Recusou (Refused)<br>◯ 2 = Não conseguiu fornecer (Did not manage to provide)<br>◯ 3 = Outro (Other)
Se Outro, especificar:<br>If Other, specify:	__________________________________
d) Suor (4)<br>d) Sweat (4)	
1. Coleta programada?<br>1. Planned for collection?	◯ 0 = Não (No)<br>◯ 1 = Sim (Yes)
2. Se programada, amostra coletada?<br>2. If due, sample collected?	◯ 0 = Não (No)<br>◯ 1 = Sim (Yes)
3. Se não, por quê?<br>3. If not collected, why?	◯ 1 = Recusou (Refused)<br>◯ 2 = Não conseguiu fornecer (Did not manage to provide)<br>◯ 3 = Outro (Other)
Se Outro, especificar:<br>If Other, specify:	__________________________________
e) Lágrimas (5)<br>e) Tears (5)	
1. Coleta programada?<br>1. Planned for collection?	◯ 0 = Não (No)<br>◯ 1 = Sim (Yes)
2. Se programada, amostra coletada?<br>2. If due, sample collected?	◯ 0 = Não (No)<br>◯ 1 = Sim (Yes)
3. Se não, por quê?<br>3. If not collected, why?	◯ 1 = Recusou (Refused)<br>◯ 2 = Não conseguiu fornecer (Did not manage to provide)<br>◯ 3 = Outro (Other)
Se Outro, especificar:<br>If Other, specify:	__________________________________
f) Fluido retal (6)<br>f) Rectal fluid (6)	
1. Coleta programada?<br>1. Planned for collection?	◯ 0 = Não (No)<br>◯ 1 = Sim (Yes)
2. Se programada, amostra coletada?<br>2. If due, sample collected?	◯ 0 = Não (No)<br>◯ 1 = Sim (Yes)

3. Se não, por quê?<br>3. If not collected, why?	◯ 1 = Recusou (Refused)<br>◯ 2 = Não conseguiu fornecer (Did not manage to provide)<br>◯ 3 = Outro (Other)
Se Outro, especificar:<br>If Other, specify:	__________________________________
g) Sêmen (7)<br>g) Semen (7)	
1. Coleta programada?<br>1. Planned for collection?	◯ 0 = Não (No)<br>◯ 1 = Sim (Yes)
2. Se programada, amostra coletada?<br>2. If due, sample collected?	◯ 0 = Não (No)<br>◯ 1 = Sim (Yes)
3. Se não, por quê?<br>3. If not collected, why?	◯ 1 = Recusou (Refused)<br>◯ 2 = Não conseguiu fornecer (Did not manage to provide)<br>◯ 3 = Outro (Other)
Se Outro, especificar:<br>If Other, specify:	__________________________________
h) Vaginal/Menstrual (8)<br>h) Vaginal/Menstrual (8)	
1. Coleta programada?<br>1. Planned for collection?	◯ 0 = Não (No)<br>◯ 1 = Sim (Yes)
2. Se programada, amostra coletada?<br>2. If due, sample collected?	◯ 0 = Não (No)<br>◯ 1 = Sim (Yes)
3. Se coletado, qual?<br>3. If collected, type?	◯ 1 = Vaginal (Vaginal)<br>◯ 2 = Menstrual (Menstrual)
4. Se não, por quê?<br>4. If not collected, why?	◯ 1 = Recusou (Refused)<br>◯ 2 = Não conseguiu fornecer (Did not manage to provide)<br>◯ 3 = Outro (Other)
Se Outro, especificar:<br>If Other, specify:	__________________________________
i) Leite materno (9) - Mama direita<br>i) Breast milk (9) - Right breast	
1. Coleta programada?<br>1. Planned for collection?	◯ 0 = Não (No)<br>◯ 1 = Sim (Yes)
2. Se programada, amostra coletada?<br>2. If due, sample collected?	◯ 0 = Não (No)<br>◯ 1 = Sim (Yes)

3. Se não, por quê?<br>3. If not collected, why?	◯ 1 = Recusou (Refused)<br>◯ 2 = Não conseguiu fornecer (Did not manage to provide)<br>◯ 3 = Outro (Other)
Se Outro, especificar:<br>If Other, specify:	__________________________________
i) Leite materno (9) - Mama esquerda<br>i) Breast milk (9) - Left breast	
1. Coleta programada?<br>1. Planned for collection?	◯ 0 = Não (No)<br>◯ 1 = Sim (Yes)
2. Se programada, amostra coletada?<br>2. If due, sample collected?	◯ 0 = Não (No)<br>◯ 1 = Sim (Yes)
3. Se não, por quê?<br>3. If not collected, why?	◯ 1 = Recusou (Refused)<br>◯ 2 = Não conseguiu fornecer (Did not manage to provide)<br>◯ 3 = Outro (Other)
Se Outro, especificar:<br>If Other, specify:	__________________________________

Observações:
Remarks:
