# Supplementary material for: Cohort profile: Study on Zika virus infection in Brazil (ZIKABRA study)
Source: PLoS One. 2021 Jan 5;16(1):e0244981. doi: 10.1371/journal.pone.0244981 (PMC7785242; doi:10.1371/journal.pone.0244981)
Supplement: S6 File — (PDF) [file pone.0244981.s006.pdf]

RES

Número de triagem: __________________________________

A65921 - Persistência do vírus Zika nos fluidos corporais de pacientes com infecção pelo vírus Zika
Questionário de Coleta de Amostras

A65921 - Persistence of Zika virus in body fluids of patients with Zika virus infection
Sample Collection Questionnaire

Número único de identificação:
Unique ID number: __________________________________

AMOSTRAS
SAMPLES

1. Sangue
1. Blood

Ct-ZIKV:
Ct-ZIKV: __________________________________
(0 = Undetermined)

INT-ZIKV:
INT-ZIKV: ◯ Detected ◯ Not Detected ◯ No Interpret

2. Urina
2. Urine

Ct-ZIKV:
Ct-ZIKV: __________________________________
(0 = Undetermined)

INT-ZIKV:
INT-ZIKV: ◯ Detected ◯ Not Detected ◯ No Interpret

3. Saliva
3. Saliva

Ct-ZIKV:
Ct-ZIKV: __________________________________
(0 = Undetermined)

INT-ZIKV:
INT-ZIKV: ◯ Detected ◯ Not Detected ◯ No Interpret

4. Suor
4. Sweat

Ct-ZIKV:<br>Ct-ZIKV:	______________________<br>(0 = Undetermined)
INT-ZIKV:<br>INT-ZIKV:	◯ Detected ◯ Not Detected<br>◯ No Interpret
5. Lágrimas<br>5. Tears	
Ct-ZIKV:<br>Ct-ZIKV:	______________________<br>(0 = Undetermined)
INT-ZIKV:<br>INT-ZIKV:	◯ Detected ◯ Not Detected<br>◯ No Interpret
6. Fluido retal<br>6. Rectal fluid	
Ct-ZIKV:<br>Ct-ZIKV:	______________________<br>(0 = Undetermined)
INT-ZIKV:<br>INT-ZIKV:	◯ Detected ◯ Not Detected<br>◯ No Interpret
7. Sêmen<br>7. Semen	
Ct-ZIKV:<br>Ct-ZIKV:	______________________<br>(0 = Undetermined)
INT-ZIKV:<br>INT-ZIKV:	◯ Detected ◯ Not Detected<br>◯ No Interpret
8. Vaginal/Menstrual<br>8. Vaginal/Menstrual	
Ct-ZIKV:<br>Ct-ZIKV:	______________________<br>(0 = Undetermined)
INT-ZIKV:<br>INT-ZIKV:	◯ Detected ◯ Not Detected<br>◯ No Interpret
9. Leite materno - Mama direita<br>9. Breast milk - Right breast	
Ct-ZIKV:<br>Ct-ZIKV:	______________________<br>(0 = Undetermined)

INT-ZIKV:
INT-ZIKV:

◯ Detected ◯ Not Detected
◯ No Interpret

9. Leite materno - Mama esquerda
9. Breast milk - Left breast

Ct-ZIKV:
Ct-ZIKV:

____
(0 = Undetermined)

INT-ZIKV:
INT-ZIKV:

◯ Detected ◯ Not Detected
◯ No Interpret
