# Supplementary material for: Cohort profile: Study on Zika virus infection in Brazil (ZIKABRA study)
Source: PLoS One. 2021 Jan 5;16(1):e0244981. doi: 10.1371/journal.pone.0244981 (PMC7785242; doi:10.1371/journal.pone.0244981)
Supplement: S7 File — (PDF) [file pone.0244981.s007.pdf]

RAP

Número de triagem: ______________________

A65921 - Persistência do vírus Zika nos fluidos corporais de pacientes com infecção pelo vírus Zika
Resultados dos testes rapidos

A65921 - Persistence of Zika virus in body fluids of patients with Zika virus infection
Rapid tests results

Centro:
Centre:

◯ 51 = Manaus - FMT
◯ 81 = Rio de Janeiro - FIOCRUZ
◯ 91 = Recife - HC

Número único de identificação:
Unique ID number: ______________________

Repetir Número único de identificação:
Repeat Unique ID number: ______________________

"Número único de identificação" e "Repetir Número único de identificação" estão diferentes, por favor verificar!

"Unique ID number" and "Repeat Unique ID number" are different, please verify!

Se Centro = 51 (Manaus - FMT), então "Número de Identificação Única" deve ser entre 151001 - 151300 ou 251001 - 251300!

If Centre = 51 (Manaus - FMT), then "Unique ID number" should be between 151001 - 151300 or 251001 - 251300!

Se Centro = 81 (Manaus - FMT), então "Número de Identificação Única" deve ser entre 181001 - 181300 ou 281001 - 281300!

If Centre = 81 (Manaus - FMT), then "Unique ID number" should be between 181001 - 181300 or 281001 - 281300!

Se Centro = 91 (Manaus - FMT), então "Número de Identificação Única" deve ser entre 191001 - 191300 ou 291001 - 291300!

If Centre = 91 (Manaus - FMT), then "Unique ID number" should be between 191001 - 191300 or 291001 - 291300!

VISITA
VISIT

1. a) Data de realização dos testes:
1. a) Date of test: ______________________

b) Iniciais do profissional que realizou o teste:
b) Initials of the professional who performed the test:

◯ JBL = João bosco
◯ MCC = Márcia
◯ MYO = Maianne

b) Iniciais do profissional que realizou o teste:
b) Initials of the professional who performed the test:

◯ ARM = Armando

2. Data de coleta de amostra:
2. Date of specimen collection: ______________________

3. Número da visita:
3. Visit number: ______________________

RESULTADOS
RESULTS

4. HIV:
4. HIV:

◯ 0 = Negativo 0 = Negative
◯ 1 = Positivo 1 = Positive
◯ 2 = Indeterminado 2 = Indeterminate
◯ 3 = Não efetuado 3 = Not performed
◯ 4 = Não aplicável 4 = Not applicable

5. Hep B:
5. Hep B:

◯ 0 = Negativo 0 = Negative
◯ 1 = Positivo 1 = Positive
◯ 2 = Indeterminado 2 = Indeterminate
◯ 3 = Não efetuado 3 = Not performed
◯ 4 = Não aplicável 4 = Not applicable

6. Hep C:
6. Hep C:

◯ 0 = Negativo 0 = Negative
◯ 1 = Positivo 1 = Positive
◯ 2 = Indeterminado 2 = Indeterminate
◯ 3 = Não efetuado 3 = Not performed
◯ 4 = Não aplicável 4 = Not applicable

7. Sifilis:
7. Syphilis:

◯ 0 = Negativo 0 = Negative
◯ 1 = Positivo 1 = Positive
◯ 2 = Indeterminado 2 = Indeterminate
◯ 3 = Não efetuado 3 = Not performed
◯ 4 = Não aplicável 4 = Not applicable

8. Gravidez:
8. Pregnancy:

◯ 0 = Negativo 0 = Negative
◯ 1 = Positivo 1 = Positive
◯ 2 = Indeterminado 2 = Indeterminate
◯ 3 = Não efetuado 3 = Not performed
◯ 4 = Não aplicável 4 = Not applicable

Observações:
Remarks:
