# Supplementary material for: Cohort profile: Study on Zika virus infection in Brazil (ZIKABRA study)
Source: PLoS One. 2021 Jan 5;16(1):e0244981. doi: 10.1371/journal.pone.0244981 (PMC7785242; doi:10.1371/journal.pone.0244981)
Supplement: S8 File — (PDF) [file pone.0244981.s008.pdf]

Confidential

CLA

Número de triagem: ________________________________

A65921 - Persistência do vírus Zika nos fluidos corporais de pacientes com infecção pelo vírus Zika
Questionário de Exame Clínico Sistemático

A65921 - Persistence of Zika virus in body fluids of patients with Zika virus infection
Systematic Medical Consultation Questionnaire

LEMBRETE: Se o CLA for preenchido nessa visita, o log de desvio também deve ser preenchido!

Centro:
Centre:

◯ 51 = Manaus - FMT
◯ 81 = Rio de Janeiro - FIOCRUZ
◯ 91 = Recife - HC

Número único de identificação:
Unique ID number: ________________________________

Repetir Número único de identificação:
Repeat Unique ID number: ________________________________

"Número de Identificação Única" e "Repetir Número de Identificação Única" estão diferentes, por favor verificar!

"Unique ID number" and "Repeat Unique ID number" are different, please verify!

Se Centro = 51 (Manaus - FMT), então "Número de Identificação Única" deve ser entre 151001 - 151300 ou 251001 - 251300!

If Centre = 51 (Manaus - FMT), then "Unique ID number" should be between 151001 - 151300 or 251001 - 251300!

Se Centro = 81 (Manaus - FMT), então "Número de Identificação Única" deve ser entre 181001 - 181300 ou 281001 - 281300!

If Centre = 81 (Manaus - FMT), then "Unique ID number" should be between 181001 - 181300 or 281001 - 281300!

Se Centro = 91 (Manaus - FMT), então "Número de Identificação Única" deve ser entre 191001 - 191300 ou 291001 - 291300!

If Centre = 91 (Manaus - FMT), then "Unique ID number" should be between 191001 - 191300 or 291001 - 291300!

INFORMAÇÃO SOBRE VISITA DE SEGUIMENTO
INFORMATION ON FOLLOW-UP VISIT

1. Data da visita:
1. Date of visit: __________________________________

2. Número da visita:
2. Visit number: __________________________________

Profissional de saúde que preencheu o questionário (iniciais):
Health professional who completed the questionnaire (Initials):

- ◯ CAB = Camila Botto
- ◯ ALA = Aline Alencar

Profissional de saúde que preencheu o questionário (iniciais):
Health professional who completed the questionnaire (Initials):

- ◯ CRB = Carlos Brito
- ◯ PAS = Paulo Sergio
- ◯ DAP = Danylo Pereira
- ◯ BRS = Braulio Silveira
- ◯ ALS = Aletheia Sampaio
- ◯ HIB = Hildenice Bernardes

COMORBIDADES
COMORBIDITIES

3. Você atualmente sofre de alguma das seguintes condições?
3. Do you currently suffer from any of the following conditions below?

	Sim (Yes)	Não (No)	Não sabe (Don't know)
a) Diabetes:<br>a) Diabetes:	◯	◯	◯
b) Hipertensão:<br>b) Hypertension:	◯	◯	◯
c) Doença articular:<br>c) Joint disease:	◯	◯	◯
d) Hepatite crônica (B ou C):<br>d) Chronic hepatitis (B or C):	◯	◯	◯
e) Malária nos últimos 30 dias:<br>e) Malaria in the last 30 days:	◯	◯	◯
f) Outras doenças crônicas por mais de 30 dias:<br>f) Other chronic disease for longer than 30 days:	◯	◯	◯

Se Sim, especificar:
If Yes, specify: __________________________________

4. a) Você já recebeu transfusão de sangue?
4. a) Have you ever received a blood transfusion?

- ◯ 0 = Não (No)
- ◯ 1 = Sim (Yes)

b) Se Sim, há quanto tempo?
b) If Yes, how long ago?

- ◯ 1 = Menos de 30 dias atrás (Less than 30 days ago)
- ◯ 2 = 30 dias ou mais atrás (30 days or longer ago)

5. Você está tomando alguma medicação regularmente (por mais de 30 dias)?<br>5. Are you taking any medication regularly (since more than 30 days)?	◯ 0 = Não (No)<br>◯ 1 = Sim (Yes)<br>◯ 8 = Não sabe (Don't know)<br>◯ 9 = Recusou (Refused)
Se Sim, especificar:<br>If Yes, specify:	__________________________________
6. Você tem alguma alergia?<br>6. Do you have any allergy?	◯ 0 = Não (No)<br>◯ 1 = Sim (Yes)<br>◯ 8 = Não sabe (Don't know)<br>◯ 9 = Recusou (Refused)
Se Sim, por favor dê detalhes:<br>If Yes, please give details:	__________________________________
7. a) Você já teve diagnóstico de Zika feito por um médico antes da infecção atual?<br>7. a) Have you ever had Zika diagnosed by a doctor before this infection?	◯ 0 = Não (No)<br>◯ 1 = Sim (Yes)<br>◯ 8 = Não sabe (Don't know)<br>◯ 9 = Recusou (Refused)
b) Se Sim, quantas vezes já ficou doente com esta doença antes?<br>b) If Yes, how many times have you been sick with this disease before?	__________________________________
c) 1. Data em que o último episódio começou:<br>c) 1. Date when the last episode started:	
Data:<br>Date:	__________________________________
Se dia desconhecido, por favor preencher "Mês" e "Ano" abaixo!<br>If day is unknown, please enter "Month" and "Year" below!	
Mês:<br>Month:	◯ Desconhecido<br>◯ Jan<br>◯ Fev<br>◯ Mar<br>◯ Abr<br>◯ Mai<br>◯ Jun<br>◯ Jul<br>◯ Ago<br>◯ Set<br>◯ Out<br>◯ Nov<br>◯ Dez

Ano:
Year:

◯ Desconhecido
◯ 1970
◯ 1971
◯ 1972
◯ 1973
◯ 1974
◯ 1975
◯ 1976
◯ 1977
◯ 1978
◯ 1979
◯ 1980
◯ 1981
◯ 1982
◯ 1983
◯ 1984
◯ 1985
◯ 1986
◯ 1987
◯ 1988
◯ 1989
◯ 1990
◯ 1991
◯ 1992
◯ 1993
◯ 1994
◯ 1995
◯ 1996
◯ 1997
◯ 1998
◯ 1999
◯ 2000
◯ 2001
◯ 2002
◯ 2003
◯ 2004
◯ 2005
◯ 2006
◯ 2007
◯ 2008
◯ 2009
◯ 2010
◯ 2011
◯ 2012
◯ 2013
◯ 2014
◯ 2015
◯ 2016
◯ 2017
◯ 2018
◯ 2019

---

2. Data em que o episódio anterior começou:
2. Date when the previous episode started:

Data:
Date: ______________________________

Se dia desconhecido, por favor preencher "Mês" e "Ano" abaixo!
If day is unknown, please enter "Month" and "Year" below!

Mês:<br>Month:	◯ Desconhecido<br>◯ Jan<br>◯ Fev<br>◯ Mar<br>◯ Abr<br>◯ Mai<br>◯ Jun<br>◯ Jul<br>◯ Ago<br>◯ Set<br>◯ Out<br>◯ Nov<br>◯ Dez

Ano:
Year:

- ◯ Desconhecido
- ◯ 1970
- ◯ 1971
- ◯ 1972
- ◯ 1973
- ◯ 1974
- ◯ 1975
- ◯ 1976
- ◯ 1977
- ◯ 1978
- ◯ 1979
- ◯ 1980
- ◯ 1981
- ◯ 1982
- ◯ 1983
- ◯ 1984
- ◯ 1985
- ◯ 1986
- ◯ 1987
- ◯ 1988
- ◯ 1989
- ◯ 1990
- ◯ 1991
- ◯ 1992
- ◯ 1993
- ◯ 1994
- ◯ 1995
- ◯ 1996
- ◯ 1997
- ◯ 1998
- ◯ 1999
- ◯ 2000
- ◯ 2001
- ◯ 2002
- ◯ 2003
- ◯ 2004
- ◯ 2005
- ◯ 2006
- ◯ 2007
- ◯ 2008
- ◯ 2009
- ◯ 2010
- ◯ 2011
- ◯ 2012
- ◯ 2013
- ◯ 2014
- ◯ 2015
- ◯ 2016
- ◯ 2017
- ◯ 2018
- ◯ 2019

---

3. Data em que o episódio anterior ao acima começou:
3. Date when the previous episode, prior to the one above, started:

Data:
Date: ______________________________

Se dia desconhecido, por favor preencher "Mês" e "Ano" abaixo!
If day is unknown, please enter "Month" and "Year" below!

Mês:<br>Month:	◯ Desconhecido<br>◯ Jan<br>◯ Fev<br>◯ Mar<br>◯ Abr<br>◯ Mai<br>◯ Jun<br>◯ Jul<br>◯ Ago<br>◯ Set<br>◯ Out<br>◯ Nov<br>◯ Dez

Ano:<br>Year:	◯ Desconhecido<br>◯ 1970<br>◯ 1971<br>◯ 1972<br>◯ 1973<br>◯ 1974<br>◯ 1975<br>◯ 1976<br>◯ 1977<br>◯ 1978<br>◯ 1979<br>◯ 1980<br>◯ 1981<br>◯ 1982<br>◯ 1983<br>◯ 1984<br>◯ 1985<br>◯ 1986<br>◯ 1987<br>◯ 1988<br>◯ 1989<br>◯ 1990<br>◯ 1991<br>◯ 1992<br>◯ 1993<br>◯ 1994<br>◯ 1995<br>◯ 1996<br>◯ 1997<br>◯ 1998<br>◯ 1999<br>◯ 2000<br>◯ 2001<br>◯ 2002<br>◯ 2003<br>◯ 2004<br>◯ 2005<br>◯ 2006<br>◯ 2007<br>◯ 2008<br>◯ 2009<br>◯ 2010<br>◯ 2011<br>◯ 2012<br>◯ 2013<br>◯ 2014<br>◯ 2015<br>◯ 2016<br>◯ 2017<br>◯ 2018<br>◯ 2019
8. a) Você já teve diagnóstico de Dengue feito por um médico antes da infecção atual?<br>8. a) Have you ever had Dengue diagnosed by a doctor before?	◯ 0 = Não (No)<br>◯ 1 = Sim (Yes)<br>◯ 8 = Não sabe (Don't know)<br>◯ 9 = Recusou (Refused)

b) Se Sim, quantas vezes já ficou doente com esta doença antes?<br>b) If Yes, how many times have you been sick with this disease before?	__________________________
c) 1. Data em que o último episódio começou:<br>c) 1. Date when the last episode started:	
Data:<br>Date:	__________________________
Se dia desconhecido, por favor preencher "Mês" e "Ano" abaixo!<br>If day is unknown, please enter "Month" and "Year" below!	
Mês:<br>Month:	◯ Desconhecido<br>◯ Jan<br>◯ Fev<br>◯ Mar<br>◯ Abr<br>◯ Mai<br>◯ Jun<br>◯ Jul<br>◯ Ago<br>◯ Set<br>◯ Out<br>◯ Nov<br>◯ Dez

Ano:<br>Year:	◯ Desconhecido<br>◯ 1970<br>◯ 1971<br>◯ 1972<br>◯ 1973<br>◯ 1974<br>◯ 1975<br>◯ 1976<br>◯ 1977<br>◯ 1978<br>◯ 1979<br>◯ 1980<br>◯ 1981<br>◯ 1982<br>◯ 1983<br>◯ 1984<br>◯ 1985<br>◯ 1986<br>◯ 1987<br>◯ 1988<br>◯ 1989<br>◯ 1990<br>◯ 1991<br>◯ 1992<br>◯ 1993<br>◯ 1994<br>◯ 1995<br>◯ 1996<br>◯ 1997<br>◯ 1998<br>◯ 1999<br>◯ 2000<br>◯ 2001<br>◯ 2002<br>◯ 2003<br>◯ 2004<br>◯ 2005<br>◯ 2006<br>◯ 2007<br>◯ 2008<br>◯ 2009<br>◯ 2010<br>◯ 2011<br>◯ 2012<br>◯ 2013<br>◯ 2014<br>◯ 2015<br>◯ 2016<br>◯ 2017<br>◯ 2018<br>◯ 2019
2. Data em que o episódio anterior começou:<br>2. Date when the previous episode started:	
Data:<br>Date:	________________________________
Se dia desconhecido, por favor preencher "Mês" e "Ano" abaixo!<br>If day is unknown, please enter "Month" and "Year" below!	

Mês:<br>Month:	◯ Desconhecido<br>◯ Jan<br>◯ Fev<br>◯ Mar<br>◯ Abr<br>◯ Mai<br>◯ Jun<br>◯ Jul<br>◯ Ago<br>◯ Set<br>◯ Out<br>◯ Nov<br>◯ Dez

Ano:
Year:

◯ Desconhecido
◯ 1970
◯ 1971
◯ 1972
◯ 1973
◯ 1974
◯ 1975
◯ 1976
◯ 1977
◯ 1978
◯ 1979
◯ 1980
◯ 1981
◯ 1982
◯ 1983
◯ 1984
◯ 1985
◯ 1986
◯ 1987
◯ 1988
◯ 1989
◯ 1990
◯ 1991
◯ 1992
◯ 1993
◯ 1994
◯ 1995
◯ 1996
◯ 1997
◯ 1998
◯ 1999
◯ 2000
◯ 2001
◯ 2002
◯ 2003
◯ 2004
◯ 2005
◯ 2006
◯ 2007
◯ 2008
◯ 2009
◯ 2010
◯ 2011
◯ 2012
◯ 2013
◯ 2014
◯ 2015
◯ 2016
◯ 2017
◯ 2018
◯ 2019

---

3. Data em que o episódio anterior ao acima começou:
3. Date when the previous episode, prior to the one above, started:

Data:
Date: ________________________________

Se dia desconhecido, por favor preencher "Mês" e "Ano" abaixo!
If day is unknown, please enter "Month" and "Year" below!

Mês:<br>Month:	◯ Desconhecido<br>◯ Jan<br>◯ Fev<br>◯ Mar<br>◯ Abr<br>◯ Mai<br>◯ Jun<br>◯ Jul<br>◯ Ago<br>◯ Set<br>◯ Out<br>◯ Nov<br>◯ Dez

Ano:<br>Year:	◯ Desconhecido<br>◯ 1970<br>◯ 1971<br>◯ 1972<br>◯ 1973<br>◯ 1974<br>◯ 1975<br>◯ 1976<br>◯ 1977<br>◯ 1978<br>◯ 1979<br>◯ 1980<br>◯ 1981<br>◯ 1982<br>◯ 1983<br>◯ 1984<br>◯ 1985<br>◯ 1986<br>◯ 1987<br>◯ 1988<br>◯ 1989<br>◯ 1990<br>◯ 1991<br>◯ 1992<br>◯ 1993<br>◯ 1994<br>◯ 1995<br>◯ 1996<br>◯ 1997<br>◯ 1998<br>◯ 1999<br>◯ 2000<br>◯ 2001<br>◯ 2002<br>◯ 2003<br>◯ 2004<br>◯ 2005<br>◯ 2006<br>◯ 2007<br>◯ 2008<br>◯ 2009<br>◯ 2010<br>◯ 2011<br>◯ 2012<br>◯ 2013<br>◯ 2014<br>◯ 2015<br>◯ 2016<br>◯ 2017<br>◯ 2018<br>◯ 2019
9. a) Você já teve diagnóstico de Chikungunya feito por um médico antes da infecção atual?<br>9. a) Have you ever had Chikungunya diagnosed by a doctor before?	◯ 0 = Não (No)<br>◯ 1 = Sim (Yes)<br>◯ 8 = Não sabe (Don't know)<br>◯ 9 = Recusou (Refused)

b) Se Sim, quantas vezes já ficou doente com esta doença antes?<br>b) If Yes, how many times have you been sick with this disease before?	__________________________
c) 1. Data em que o último episódio começou:<br>c) 1. Date when the last episode started:	
Data:<br>Date:	__________________________
Se dia desconhecido, por favor preencher "Mês" e "Ano" abaixo!<br>If day is unknown, please enter "Month" and "Year" below!	
Mês:<br>Month:	◯ Desconhecido<br>◯ Jan<br>◯ Fev<br>◯ Mar<br>◯ Abr<br>◯ Mai<br>◯ Jun<br>◯ Jul<br>◯ Ago<br>◯ Set<br>◯ Out<br>◯ Nov<br>◯ Dez

Ano:
Year:

◯ Desconhecido
◯ 1970
◯ 1971
◯ 1972
◯ 1973
◯ 1974
◯ 1975
◯ 1976
◯ 1977
◯ 1978
◯ 1979
◯ 1980
◯ 1981
◯ 1982
◯ 1983
◯ 1984
◯ 1985
◯ 1986
◯ 1987
◯ 1988
◯ 1989
◯ 1990
◯ 1991
◯ 1992
◯ 1993
◯ 1994
◯ 1995
◯ 1996
◯ 1997
◯ 1998
◯ 1999
◯ 2000
◯ 2001
◯ 2002
◯ 2003
◯ 2004
◯ 2005
◯ 2006
◯ 2007
◯ 2008
◯ 2009
◯ 2010
◯ 2011
◯ 2012
◯ 2013
◯ 2014
◯ 2015
◯ 2016
◯ 2017
◯ 2018
◯ 2019

2. Data em que o episódio anterior começou:
2. Date when the previous episode started:

Data:
Date: ______________________________

Se dia desconhecido, por favor preencher "Mês" e "Ano" abaixo!
If day is unknown, please enter "Month" and "Year" below!

Mês:<br>Month:	◯ Desconhecido<br>◯ Jan<br>◯ Fev<br>◯ Mar<br>◯ Abr<br>◯ Mai<br>◯ Jun<br>◯ Jul<br>◯ Ago<br>◯ Set<br>◯ Out<br>◯ Nov<br>◯ Dez

Ano:<br>Year:	◯ Desconhecido<br>◯ 1970<br>◯ 1971<br>◯ 1972<br>◯ 1973<br>◯ 1974<br>◯ 1975<br>◯ 1976<br>◯ 1977<br>◯ 1978<br>◯ 1979<br>◯ 1980<br>◯ 1981<br>◯ 1982<br>◯ 1983<br>◯ 1984<br>◯ 1985<br>◯ 1986<br>◯ 1987<br>◯ 1988<br>◯ 1989<br>◯ 1990<br>◯ 1991<br>◯ 1992<br>◯ 1993<br>◯ 1994<br>◯ 1995<br>◯ 1996<br>◯ 1997<br>◯ 1998<br>◯ 1999<br>◯ 2000<br>◯ 2001<br>◯ 2002<br>◯ 2003<br>◯ 2004<br>◯ 2005<br>◯ 2006<br>◯ 2007<br>◯ 2008<br>◯ 2009<br>◯ 2010<br>◯ 2011<br>◯ 2012<br>◯ 2013<br>◯ 2014<br>◯ 2015<br>◯ 2016<br>◯ 2017<br>◯ 2018<br>◯ 2019
3. Data em que o episódio anterior ao acima começou:<br>3. Date when the previous episode, prior to the one above, started:	
Data:<br>Date:	________________________________
Se dia desconhecido, por favor preencher "Mês" e "Ano" abaixo!<br>If day is unknown, please enter "Month" and "Year" below!	

Mês:<br>Month:	◯ Desconhecido<br>◯ Jan<br>◯ Fev<br>◯ Mar<br>◯ Abr<br>◯ Mai<br>◯ Jun<br>◯ Jul<br>◯ Ago<br>◯ Set<br>◯ Out<br>◯ Nov<br>◯ Dez

Ano:
Year:

◯ Desconhecido
◯ 1970
◯ 1971
◯ 1972
◯ 1973
◯ 1974
◯ 1975
◯ 1976
◯ 1977
◯ 1978
◯ 1979
◯ 1980
◯ 1981
◯ 1982
◯ 1983
◯ 1984
◯ 1985
◯ 1986
◯ 1987
◯ 1988
◯ 1989
◯ 1990
◯ 1991
◯ 1992
◯ 1993
◯ 1994
◯ 1995
◯ 1996
◯ 1997
◯ 1998
◯ 1999
◯ 2000
◯ 2001
◯ 2002
◯ 2003
◯ 2004
◯ 2005
◯ 2006
◯ 2007
◯ 2008
◯ 2009
◯ 2010
◯ 2011
◯ 2012
◯ 2013
◯ 2014
◯ 2015
◯ 2016
◯ 2017
◯ 2018
◯ 2019

---

10. a) Você já teve diagnóstico de Febre Amarela feito por um médico antes da infecção atual?
10. a) Have you ever had Yellow Fever diagnosed by a doctor before?

◯ 0 = Não (No)
◯ 1 = Sim (Yes)
◯ 8 = Não sabe (Don't know)
◯ 9 = Recusou (Refused)

b) Se Sim, quantas vezes já ficou doente com esta doença antes?
b) If Yes, how many times have you been sick with this disease before?

____

c) 1. Data em que o último episódio começou:
c) 1. Date when the last episode started:

Data:
Date:

____

Se dia desconhecido, por favor preencher "Mês" e "Ano" abaixo!
If day is unknown, please enter "Month" and "Year" below!

Mês:
Month:

- ◯ Desconhecido
- ◯ Jan
- ◯ Fev
- ◯ Mar
- ◯ Abr
- ◯ Mai
- ◯ Jun
- ◯ Jul
- ◯ Ago
- ◯ Set
- ◯ Out
- ◯ Nov
- ◯ Dez

Ano:
Year:

◯ Desconhecido
◯ 1970
◯ 1971
◯ 1972
◯ 1973
◯ 1974
◯ 1975
◯ 1976
◯ 1977
◯ 1978
◯ 1979
◯ 1980
◯ 1981
◯ 1982
◯ 1983
◯ 1984
◯ 1985
◯ 1986
◯ 1987
◯ 1988
◯ 1989
◯ 1990
◯ 1991
◯ 1992
◯ 1993
◯ 1994
◯ 1995
◯ 1996
◯ 1997
◯ 1998
◯ 1999
◯ 2000
◯ 2001
◯ 2002
◯ 2003
◯ 2004
◯ 2005
◯ 2006
◯ 2007
◯ 2008
◯ 2009
◯ 2010
◯ 2011
◯ 2012
◯ 2013
◯ 2014
◯ 2015
◯ 2016
◯ 2017
◯ 2018
◯ 2019

---

2. Data em que o episódio anterior começou:
2. Date when the previous episode started:

Data:
Date: ______________________________

Se dia desconhecido, por favor preencher "Mês" e "Ano" abaixo!
If day is unknown, please enter "Month" and "Year" below!

Mês:<br>Month:	◯ Desconhecido<br>◯ Jan<br>◯ Fev<br>◯ Mar<br>◯ Abr<br>◯ Mai<br>◯ Jun<br>◯ Jul<br>◯ Ago<br>◯ Set<br>◯ Out<br>◯ Nov<br>◯ Dez

Ano:
Year:

◯ Desconhecido
◯ 1970
◯ 1971
◯ 1972
◯ 1973
◯ 1974
◯ 1975
◯ 1976
◯ 1977
◯ 1978
◯ 1979
◯ 1980
◯ 1981
◯ 1982
◯ 1983
◯ 1984
◯ 1985
◯ 1986
◯ 1987
◯ 1988
◯ 1989
◯ 1990
◯ 1991
◯ 1992
◯ 1993
◯ 1994
◯ 1995
◯ 1996
◯ 1997
◯ 1998
◯ 1999
◯ 2000
◯ 2001
◯ 2002
◯ 2003
◯ 2004
◯ 2005
◯ 2006
◯ 2007
◯ 2008
◯ 2009
◯ 2010
◯ 2011
◯ 2012
◯ 2013
◯ 2014
◯ 2015
◯ 2016
◯ 2017
◯ 2018
◯ 2019

---

3. Data em que o episódio anterior ao acima começou:
3. Date when the previous episode, prior to the one above, started:

Data:
Date: ________________________________

Se dia desconhecido, por favor preencher "Mês" e "Ano" abaixo!
If day is unknown, please enter "Month" and "Year" below!

Mês:<br>Month:	◯ Desconhecido<br>◯ Jan<br>◯ Fev<br>◯ Mar<br>◯ Abr<br>◯ Mai<br>◯ Jun<br>◯ Jul<br>◯ Ago<br>◯ Set<br>◯ Out<br>◯ Nov<br>◯ Dez

Ano:<br>Year:	◯ Desconhecido<br>◯ 1970<br>◯ 1971<br>◯ 1972<br>◯ 1973<br>◯ 1974<br>◯ 1975<br>◯ 1976<br>◯ 1977<br>◯ 1978<br>◯ 1979<br>◯ 1980<br>◯ 1981<br>◯ 1982<br>◯ 1983<br>◯ 1984<br>◯ 1985<br>◯ 1986<br>◯ 1987<br>◯ 1988<br>◯ 1989<br>◯ 1990<br>◯ 1991<br>◯ 1992<br>◯ 1993<br>◯ 1994<br>◯ 1995<br>◯ 1996<br>◯ 1997<br>◯ 1998<br>◯ 1999<br>◯ 2000<br>◯ 2001<br>◯ 2002<br>◯ 2003<br>◯ 2004<br>◯ 2005<br>◯ 2006<br>◯ 2007<br>◯ 2008<br>◯ 2009<br>◯ 2010<br>◯ 2011<br>◯ 2012<br>◯ 2013<br>◯ 2014<br>◯ 2015<br>◯ 2016<br>◯ 2017<br>◯ 2018<br>◯ 2019
11. a) 1. Você recebeu vacina contra a Febre Amarela?<br>11. a) 1. Have you received a vaccine against Yellow Fever?	◯ 0 = Não (No)<br>◯ 1 = Sim (Yes)<br>◯ 8 = Não sabe (Don't know)<br>◯ 9 = Recusou (Refused)
2. Se Sim, data da ultima vacinação:<br>2. If Yes, date:	______________________

Se dia desconhecido, por favor preencher "Mês" e "Ano" abaixo!
If day is unknown, please enter "Month" and "Year" below!

Mês:<br>Month:	◯ Desconhecido<br>◯ Jan<br>◯ Fev<br>◯ Mar<br>◯ Abr<br>◯ Mai<br>◯ Jun<br>◯ Jul<br>◯ Ago<br>◯ Set<br>◯ Out<br>◯ Nov<br>◯ Dez

Ano:<br>Year:	◯ Desconhecido<br>◯ 1970<br>◯ 1971<br>◯ 1972<br>◯ 1973<br>◯ 1974<br>◯ 1975<br>◯ 1976<br>◯ 1977<br>◯ 1978<br>◯ 1979<br>◯ 1980<br>◯ 1981<br>◯ 1982<br>◯ 1983<br>◯ 1984<br>◯ 1985<br>◯ 1986<br>◯ 1987<br>◯ 1988<br>◯ 1989<br>◯ 1990<br>◯ 1991<br>◯ 1992<br>◯ 1993<br>◯ 1994<br>◯ 1995<br>◯ 1996<br>◯ 1997<br>◯ 1998<br>◯ 1999<br>◯ 2000<br>◯ 2001<br>◯ 2002<br>◯ 2003<br>◯ 2004<br>◯ 2005<br>◯ 2006<br>◯ 2007<br>◯ 2008<br>◯ 2009<br>◯ 2010<br>◯ 2011<br>◯ 2012<br>◯ 2013<br>◯ 2014<br>◯ 2015<br>◯ 2016<br>◯ 2017<br>◯ 2018<br>◯ 2019
b) 1. Você recebeu vacina contra a Dengue?<br>b) 1. Have you received a vaccine against DEngue?	◯ 0 = Não (No)<br>◯ 1 = Sim (Yes)<br>◯ 8 = Não sabe (Don't know)<br>◯ 9 = Recusou (Refused)
2. Se Sim, data da ultima vacinação:<br>2. If Yes, date:	______________________________
Se dia desconhecido, por favor preencher "Mês" e "Ano" abaixo!<br>If day is unknown, please enter "Month" and "Year" below!	

Mês:<br>Month:	◯ Desconhecido<br>◯ Jan<br>◯ Fev<br>◯ Mar<br>◯ Abr<br>◯ Mai<br>◯ Jun<br>◯ Jul<br>◯ Ago<br>◯ Set<br>◯ Out<br>◯ Nov<br>◯ Dez

Ano:<br>Year:	◯ Desconhecido<br>◯ 1970<br>◯ 1971<br>◯ 1972<br>◯ 1973<br>◯ 1974<br>◯ 1975<br>◯ 1976<br>◯ 1977<br>◯ 1978<br>◯ 1979<br>◯ 1980<br>◯ 1981<br>◯ 1982<br>◯ 1983<br>◯ 1984<br>◯ 1985<br>◯ 1986<br>◯ 1987<br>◯ 1988<br>◯ 1989<br>◯ 1990<br>◯ 1991<br>◯ 1992<br>◯ 1993<br>◯ 1994<br>◯ 1995<br>◯ 1996<br>◯ 1997<br>◯ 1998<br>◯ 1999<br>◯ 2000<br>◯ 2001<br>◯ 2002<br>◯ 2003<br>◯ 2004<br>◯ 2005<br>◯ 2006<br>◯ 2007<br>◯ 2008<br>◯ 2009<br>◯ 2010<br>◯ 2011<br>◯ 2012<br>◯ 2013<br>◯ 2014<br>◯ 2015<br>◯ 2016<br>◯ 2017<br>◯ 2018<br>◯ 2019
c) 1. Você recebeu outras vacinas na fase adulta?<br>c) Have you received any other vaccine during adulthood?	◯ 0 = Não (No)<br>◯ 1 = Sim (Yes)<br>◯ 8 = Não sabe (Don't know)<br>◯ 9 = Recusou (Refused)

2. Se Sim, especifique quais vacinas:
2. If Yes, report which vaccines:

☐ 1 = Hepatite A (Hepatitis A)
☐ 2 = Hepatite B (Hepatitis B)
☐ 3 = Sarampo (Measles)
☐ 4 = Rubéola (Rubella)
☐ 5 = Tétano (Tetanus)
☐ 6 = Difteria (Diphtheria)
☐ 7 = Gripe (Flue)
☐ 8 = HPV (HPV)
☐ 9 = Outras, desconhecidas (Other, unknown)
☐ 10 = Outras, conhecidas (Other, known)

Se Outras conhecidas, especificar:
If Other known, specify: ______________________

---

d) Você ja participou de alguma pesquisa clínica para vacina?
d) Have you already participated in any clinical research on immunization?

○ 0 = Não (No)
○ 1 = Sim (Yes)
○ 8 = Não sabe (Don't know)
○ 9 = Recusou (Refused)

Se Sim, especifique:
If Yes, specify: ______________________

PENDENTE DESDE A ÚLTIMA VISITA
PENDING SINCE LAST VISIT

12. a) Algum novo resultado de exames?
12. a) Any new result of investigations?

○ 0 = Não (No)
○ 1 = Sim (Yes)

---

b) Resultado do exame 1:
b) Result of investigation 1:

---

1. Especifique a investigação:
1. Specify the investigation: ______________________

---

2. Resultado:
2. Result: ______________________

---

3. Outro novo resultado de exame disponível?
3. Other available result of investigation?

○ 0 = Não (No)
○ 1 = Sim (Yes)

---

c) Resultado do exame 2:
c) Result of investigation 2:

---

1. Especifique a investigação:
1. Specify the investigation: ______________________

2. Resultado:
2. Result: ________________________________

3. Outro novo resultado de exame disponível?
3. Other available result of investigation?
- ◯ 0 = Não (No)
- ◯ 1 = Sim (Yes)

d) Resultado do exame 3:
d) Result of investigation 3:

1. Especifique a investigação:
1. Specify the investigation: ________________________________

2. Resultado:
2. Result: ________________________________

13. a) Algum novo resultado de encaminhamento a especialista?
13. a) Any new result of referral to a specialist?
- ◯ 0 = Não (No)
- ◯ 1 = Sim (Yes)

b) Resultado de encaminhamento a especialista 1:
b) Result of referral to specialist 1:

1. Especifique o especialista:
1. Specify the specialist: ________________________________

2. Resultado:
2. Result: ________________________________

3. Outro novo resultado de encaminhamento a um especialista disponível?
3. Other available result of referal to a specialist?
- ◯ 0 = Não (No)
- ◯ 1 = Sim (Yes)

c) Resultado de encaminhamento a especialista 2:
c) Result of referral to specialist 2:

1. Especifique o especialista:
1. Specify the specialist: ________________________________

2. Resultado:
2. Result: ________________________________

14. Sintomas que ocorreram... desde o início deste episódio (V01-Sintomático)
... desde as últimas três semanas (V01-Assintomático)
... desde as últimas três semanas (V05)
... desde a última visita (V17)14. Symptoms that occurred... since the initial phase of this episode (V01-Symptomatic)
... during the last 3 weeks (V01-Asymptomatic)
... during the last 3 weeks (V05)
... since last visit (V17)

a) Gerais:
a) General

	Não (No)	Sim (Yes)
1. Febre<br>1. Fever	◯	◯
2. Calafrios<br>2. Chills	◯	◯
3. Sudorese<br>3. Sweat	◯	◯
4. Icterícia<br>4. Jaundice	◯	◯
5. Sangramento<br>5. Bleeding	◯	◯

Se Sangramento presente durante esse tempo, indique o(s) tipo(s) presente(s):
If Bleeding present during this period, specify which type(s) is/are present:

	Não (No)	Sim (Yes)
a) Epistaxe<br>a) Epistaxis	◯	◯
b) Gengival<br>b) Gingival	◯	◯
c) Metrorragia<br>c) Metrorrhagia	◯	◯
d) Hematúria<br>d) Hematuria	◯	◯
e) Hematêmese<br>e) Hematemesis	◯	◯
f) Melena<br>f) Melena	◯	◯
g) Hemoptise<br>g) Hemoptysis	◯	◯
h) Outro<br>h) Other	◯	◯

Se Outro, especifique:
If Other, specify: __________________________________

b) Dermatológicos:
b) Dermatological:

1. Exantema:
1. Rash:
◯ 0 = Não (No)
◯ 1 = Sim (Yes)

Se Exantema presente durante esse tempo, indique os tipos presente e o modo de expansão
If Rash present during this period specify which type and how it spread

a) 1. Exantema macular presente?
a) 1. Macular rash present?
◯ 0 = Não (No)
◯ 1 = Sim (Yes)

2. Se Presente, expansão:
2. If Present, how did it spread:
◯ 1 = Centrifugal (Centrifugal)
◯ 2 = Centripetal (Centripetal)
◯ 9 = Não sabe (Don't know)

b) 1. Exantema maculopapular presente?
b) 1. Maculopapular rash present?
◯ 0 = Não (No)
◯ 1 = Sim (Yes)

2. Se Presente, expansão:<br>2. If Present, how did it spread:	◯ 1 = Centrifugal (Centrifugal)<br>◯ 2 = Centripetal (Centripetal)<br>◯ 9 = Não sabe (Don't know)
c) 1. Eritema presente?<br>c) 1. Erythema present?	◯ 0 = Não (No)<br>◯ 1 = Sim (Yes)
2. Se Presente, expansão:<br>2. If Present, how did it spread:	◯ 1 = Centrifugal (Centrifugal)<br>◯ 2 = Centripetal (Centripetal)<br>◯ 9 = Não sabe (Don't know)
d) 1. Exantema vesicular presente?<br>d) 1. Vesicular rash present?	◯ 0 = Não (No)<br>◯ 1 = Sim (Yes)
2. Se Presente, expansão:<br>2. If Present, how did it spread:	◯ 1 = Centrifugal (Centrifugal)<br>◯ 2 = Centripetal (Centripetal)<br>◯ 9 = Não sabe (Don't know)
e) 1. Petéquia ou púrpura presente?<br>e) 1. Petechiae or purpura present?	◯ 0 = Não (No)<br>◯ 1 = Sim (Yes)
2. Se Presente, expansão:<br>2. If Present, how did it spread:	◯ 1 = Centrifugal (Centrifugal)<br>◯ 2 = Centripetal (Centripetal)<br>◯ 9 = Não sabe (Don't know)
f) 1. Equimoses presente?<br>f) 1. Ecchymosis present?	◯ 0 = Não (No)<br>◯ 1 = Sim (Yes)
2. Se Presente, expansão:<br>2. If Present, how did it spread:	◯ 1 = Centrifugal (Centrifugal)<br>◯ 2 = Centripetal (Centripetal)<br>◯ 9 = Não sabe (Don't know)
g) 1. Outro presente?<br>g) 1. Other present?	◯ 0 = Não (No)<br>◯ 1 = Sim (Yes)
Se Outro, especifique:<br>If Other, specify:	__________________________________
2. Se Presente, expansão:<br>2. If Present, how did it spread:	◯ 1 = Centrifugal (Centrifugal)<br>◯ 2 = Centripetal (Centripetal)<br>◯ 9 = Não sabe (Don't know)
2. Coceira na pele (prurido):<br>2. Skin itching (pruritus):	◯ 0 = Não (No)<br>◯ 1 = Sim (Yes)

3. Sinal de picadas de insetos:<br>3. Signs of insect bites:	◯ 0 = Não (No)<br>◯ 1 = Sim (Yes)
c) Neurológicos:<br>c) Neurological:	
1. Gerais:<br>1. General:	
a) Convulsões:<br>a) Convulsions:	◯ 0 = Não (No)<br>◯ 1 = Sim (Yes)
b) Rigidez de nuca:<br>b) Stiff neck:	◯ 0 = Não (No)<br>◯ 1 = Sim (Yes)
c) Dor de cabeça:<br>c) Headache:	◯ 0 = Não (No)<br>◯ 1 = Sim (Yes)
d) Fotofobia:<br>d) Photophobia:	◯ 0 = Não (No)<br>◯ 1 = Sim (Yes)
2. Sintomas motores:<br>2. Motor symptoms:	
a) Fraqueza muscular:<br>a) Muscular weakness:	
1. Presente?<br>1. Present?	◯ 0 = Não (No)<br>◯ 1 = Sim (Yes)
2. Se Presente, tipo:<br>2. If Present, which type:	◯ 1 = Geral (General)<br>◯ 2 = Focal (Focal)
3. Se focal, partes do corpo afetadas e especifique se quadro progressivo:<br>3. If focal, describe the parts of the body affected and the way the symptoms progressed:	______________________
b) Perda de movimento (paralisia):<br>b) Loss of movement (paralysis):	
1. Presente?<br>1. Present?	◯ 0 = Não (No)<br>◯ 1 = Sim (Yes)
2. Se Presente, tipo:<br>2. If Present, which type:	◯ 1 = Geral (General)<br>◯ 2 = Ascendente (Ascending)

3. Descreva as partes do corpo afetadas e se quadro progressivo:
3. Describe the parts of the body affected and the way the symptoms progressed:

____

c) Outro:
c) Other:

◯ 0 = Não (No)
◯ 1 = Sim (Yes)

Se Outro, especifique:
If Other, specify:

____

3. Sintomas sensitivos:
3. Sensitivity symptoms:

a) Dormência:
a) Sensation of numbness:

◯ 0 = Não (No)
◯ 1 = Sim (Yes)

b) Queimação:
b) Burning:

◯ 0 = Não (No)
◯ 1 = Sim (Yes)

c) Formigamento:
c) Tingling:

◯ 0 = Não (No)
◯ 1 = Sim (Yes)

d) Outro:
d) Other:

◯ 0 = Não (No)
◯ 1 = Sim (Yes)

Se Outro, especifique:
If Other, specify:

____

4. Alteração do comportamento:
4. Modification of behaviour:

a) Irritabilidade:
a) Irritability:

◯ 0 = Não (No)
◯ 1 = Sim (Yes)

b) Agitação:
b) Agitation:

◯ 0 = Não (No)
◯ 1 = Sim (Yes)

c) Sonolência:
c) Drowsiness:

◯ 0 = Não (No)
◯ 1 = Sim (Yes)

d) Prostração:
d) Prostration:

◯ 0 = Não (No)
◯ 1 = Sim (Yes)

e) Confusão/desorientação:<br>e) Confusion/disorientation:	◯ 0 = Não (No)<br>◯ 1 = Sim (Yes)
f) Amnésia:<br>f) Amnesia:	◯ 0 = Não (No)<br>◯ 1 = Sim (Yes)
g) Depressão:<br>g) Depression:	◯ 0 = Não (No)<br>◯ 1 = Sim (Yes)
h) Outro:<br>h) Other:	◯ 0 = Não (No)<br>◯ 1 = Sim (Yes)
Se Outro, especifique:<br>If Other, specify:	__________________________________
5. Incoordenação motora:<br>5. Lack of coordination in movements:	
a) Falta de precisão na coordenação dos movimentos:<br>a) Lack of precision of coordination in movements:	◯ 0 = Não (No)<br>◯ 1 = Sim (Yes)
b) Alterações do equilíbrio:<br>b) Lack of balance:	◯ 0 = Não (No)<br>◯ 1 = Sim (Yes)
c) Outro:<br>c) Other:	◯ 0 = Não (No)<br>◯ 1 = Sim (Yes)
Se Outro, especifique:<br>If Other, specify:	__________________________________
6. Comprometimento dos nervos cranianos:<br>6. Cranial nerves impairment:	
a) Alterção da visão:<br>a) Sight impairment:	◯ 0 = Não (No)<br>◯ 1 = Sim (Yes)
b) Alteração do olfato:<br>b) Smell impairment:	◯ 0 = Não (No)<br>◯ 1 = Sim (Yes)
c) Alteração do paladar:<br>c) Taste impairment:	◯ 0 = Não (No)<br>◯ 1 = Sim (Yes)

d) Alterações dos movimento dos olhos:<br>d) Eyes movements' impairment:	◯ 0 = Não (No)<br>◯ 1 = Sim (Yes)
e) Alterações dos movimentos da língua:<br>e) Tongue movements' impairment:	◯ 0 = Não (No)<br>◯ 1 = Sim (Yes)
f) Alterações dos movimento dos ombros:<br>f) Shoulders movements' impairment:	◯ 0 = Não (No)<br>◯ 1 = Sim (Yes)
g) Alterações dos movimento da face:<br>g) Face movements' impairment:	◯ 0 = Não (No)<br>◯ 1 = Sim (Yes)
h) Outro:<br>h) Other:	◯ 0 = Não (No)<br>◯ 1 = Sim (Yes)
Se Outro, especifique:<br>If Other, specify:	________________________
d) Cabeça:<br>d) Head:	
1. Coriza:<br>1. Coryza:	◯ 0 = Não (No)<br>◯ 1 = Sim (Yes)
2. Congestão nasal:<br>2. Running nose:	◯ 0 = Não (No)<br>◯ 1 = Sim (Yes)
3. Dor orofaríngea:<br>3. Oropharyngeal pain:	◯ 0 = Não (No)<br>◯ 1 = Sim (Yes)
4. Rouquidão:<br>4. Hoarseness:	◯ 0 = Não (No)<br>◯ 1 = Sim (Yes)
5. Úlceras na boca:<br>5. Mouth ulcers:	◯ 0 = Não (No)<br>◯ 1 = Sim (Yes)
6. Vermelhidão nos olhos:<br>6. Red eyes:	◯ 0 = Não (No)<br>◯ 1 = Sim (Yes)
7. Dor retro-orbitária:<br>7. Retro-orbital pain:	◯ 0 = Não (No)<br>◯ 1 = Sim (Yes)

8. Dor de ouvido:<br>8. Ear pain:	◯ 0 = Não (No)<br>◯ 1 = Sim (Yes)
9. Outro:<br>9. Other:	◯ 0 = Não (No)<br>◯ 1 = Sim (Yes)
Se Outro, especifique:<br>If Other, specify:	__________________________________
e) Respiratório/Cardiovascular:<br>e) Respiratory/Cardiovascular:	
1. Tosse:<br>1. Cough:	◯ 0 = Não (No)<br>◯ 1 = Sim (Yes)
2. Dispnéia:<br>2. Dyspnea:	◯ 0 = Não (No)<br>◯ 1 = Sim (Yes)
3. Dor no peito:<br>3. Chest pain:	◯ 0 = Não (No)<br>◯ 1 = Sim (Yes)
4. Outro:<br>4. Other:	◯ 0 = Não (No)<br>◯ 1 = Sim (Yes)
Se Outro, especifique:<br>If Other, specify:	__________________________________
f) Digestivo/Urinário:<br>f) Digestive/Urinary tract:	
1. Dor abdominal:<br>1. Abdominal pain:	◯ 0 = Não (No)<br>◯ 1 = Sim (Yes)
2. Perda de apetite/anorexia:<br>2. Loss of appetite/anorexia:	◯ 0 = Não (No)<br>◯ 1 = Sim (Yes)
3. Vômito:<br>3. Vomiting:	◯ 0 = Não (No)<br>◯ 1 = Sim (Yes)
4. Náusea:<br>4. Nausea:	◯ 0 = Não (No)<br>◯ 1 = Sim (Yes)
5. Diarréia:<br>5. Diarrhea:	◯ 0 = Não (No)<br>◯ 1 = Sim (Yes)

6. Disúria:<br>6. Dysuria:	◯ 0 = Não (No)<br>◯ 1 = Sim (Yes)
7. Colúria:<br>7. Choluria:	◯ 0 = Não (No)<br>◯ 1 = Sim (Yes)
8. Outro:<br>8. Other:	◯ 0 = Não (No)<br>◯ 1 = Sim (Yes)
Se Outro, especifique:<br>If Other, specify:	__________________________________
g) Reumatológico e músculos:<br>g) Joints and muscles:	
1. Dor nas articulações:<br>1. Joint pain:	◯ 0 = Não (No)<br>◯ 1 = Sim, apenas em uma articulação (Yes, in one joint only)<br>◯ 2 = Sim, em duas articulações ou mais (Yes, in two joints or more)
2. Edema periarticular:<br>2. Periarticular edema:	◯ 0 = Não (No)<br>◯ 1 = Sim (Yes)
3. Mialgia:<br>3. Myalgia:	◯ 0 = Não (No)<br>◯ 1 = Sim (Yes)
4. Outro:<br>4. Other:	◯ 0 = Não (No)<br>◯ 1 = Sim (Yes)
Se Outro, especifique:<br>If Other, specify:	__________________________________

EXAME FÍSICO
EXAMINATION

Informe os sinais que estão presentes hoje

15. Geral:<br>15. General signs:	☐ 0 = Nenhum (None)<br>☐ 1 = Desidratação (Dehydration)<br>☐ 2 = Edema (Edema)<br>☐ 3 = Palidez cutânea/mucosa (Pale skin/mucosa)<br>☐ 4 = Icterícia (Jaundice)

Se qualquer sinal foi selecionado, então "NENHUM" não pode ser selecionado!

If any sign is selected, then "NONE" cannot be selected!

16. Cutâneo ou da mucosa/garganta:
16. Skin and mucosa/throat signs:

- ☐ 0 = Nenhum (None)
- ☐ 1 = Exantema macular (Macular rash)
- ☐ 2 = Exantema maculopapular (Maculopapular rash)
- ☐ 3 = Eritema (Erythema)
- ☐ 4 = Exantema vesicular (Vesicular rash)
- ☐ 5 = Petéquia ou púrpura (Petechiae or purpura)
- ☐ 6 = Equimoses (Ecchymosis)
- ☐ 7 = Enantema (Enanthema)
- ☐ 8 = Hiperemia de orofaringe (Oral/pharyngeal redness)
- ☐ 9 = Úlceras na boca (Mouth ulcers)
- ☐ 10 = Outro (Other)

Se Outro, especifique:
If Other, specify: ______________________________

Se qualquer sinal foi selecionado, então "NENHUM" não pode ser selecionado!

If any sign is selected, then "NONE" cannot be selected!

---

17. Neurológico:
17. Neurological:

- ☐ 0 = Nenhum (None)
- ☐ 1 = Rigidez do pescoço (Neck stiffness)
- ☐ 2 = Fraqueza muscular (Muscular weakness)
- ☐ 3 = Paralisia (Paralysis)
- ☐ 4 = Alterações dos movimento dos olhos (Eyes movements impairment)
- ☐ 5 = Alterações dos movimentos da língua (Tongue movements impairment)
- ☐ 6 = Alterações dos movimentos dos ombros (Shoulders movements impairment)
- ☐ 7 = Alterações dos movimentos da face (Face movements impairment)
- ☐ 8 = Outro (Other)

Se Qualquer sinal neurológico presente, especifique:
If Any neurological symptoms present, please describe: ______________________________

Se qualquer sinal foi selecionado, então "NENHUM" não pode ser selecionado!

If any sign is selected, then "NONE" cannot be selected!

---

18. Sistema respiratório/cardiovascular:
18. Respiratory/cardiac abnormalities:

- ☐ 0 = Nenhum (None)
- ☐ 1 = Dispnéia (Dyspnea)
- ☐ 2 = Sopro (Murmur)
- ☐ 3 = Crepitações (Fine crackles)
- ☐ 4 = Roncos (Coarse crackles)
- ☐ 5 = Sibilos (Wheezes)
- ☐ 6 = Outro (Other)

Se Outro, especifique:
If Other, specify: __________________________________

Se qualquer sinal foi selecionado, então "NENHUM" não pode ser selecionado!

If any sign is selected, then "NONE" cannot be selected!

19. Abdominal:
19. Abdominal abnormalities:

- ☐ 0 = Nenhum (None)
- ☐ 1 = Dor à palpação (Pain on palpation)
- ☐ 2 = Ascite (Ascites)
- ☐ 3 = Hepatomegalia (Hepatomegaly)
- ☐ 4 = Esplenomegalia (Splenomegaly)
- ☐ 5 = Outro (Other)

Se Outro, especifique:
If Other, specify: __________________________________

Se qualquer sinal foi selecionado, então "NENHUM" não pode ser selecionado!

If any sign is selected, then "NONE" cannot be selected!

20. Reumatológico:
20. Joints abnormalities:

- ☐ 0 = Nenhum (None)
- ☐ 1 = Edema periarticular (Periarticular edema)
- ☐ 2 = Calor (Hotness)
- ☐ 3 = Vermelhidão (Redness)
- ☐ 4 = Outro (Other)

Se Outro, especifique:
If Other, specify: __________________________________

Se qualquer sinal foi selecionado, então "NENHUM" não pode ser selecionado!

If any sign is selected, then "NONE" cannot be selected!

21. Cadeia ganglionar:
21. Lymphadenopathy:

- ☐ 0 = Nenhum (None)
- ☐ 1 = Cervical (Cervical)
- ☐ 2 = Retro auricular (Retro auricular)
- ☐ 3 = Occipital (Occipital)
- ☐ 4 = Supraclavicular (Supraclavicular)
- ☐ 5 = Axilar (Axillar)
- ☐ 6 = Epitroclear (Epitrochlear)
- ☐ 7 = Inguinal (Inguinal)
- ☐ 8 = Outro (Other)

Se Outro, especifique:
If Other, specify: __________________________________

Se qualquer sinal foi selecionado, então "NENHUM" não pode ser selecionado!

If any sign is selected, then "NONE" cannot be selected!

---

22. a) Alguma patologia presente... desde o início deste episódio (V01-Sintomático)
... desde as últimas três semanas (V01-Assintomático)
... desde as últimas três semanas (V05)
... desde a última visita (V17)22. a) Any pathology present... since the initial phase of this episode (V01-Symptomatic)
... during the last 3 weeks (V01-Asymptomatic)
... during the last 3 weeks (V05)
... since last visit (V17)

◯ 0 = Não (No)
◯ 1 = Sim (Yes)

---

b) Para cada uma das patologias presentes neste intervalo, forneça a informação solicitada:
b) For each pathology present during this period, please provide the information requested:

Marque patologia no Checklist!

Check Pathology on Checklist!

---

1. Patologia 1:
1. Pathology 1:

---

a) Especifique:
a) Specify: __________________________________

---

b) Presente hoje?
b) Present today?

◯ 0 = Não (No)
◯ 1 = Sim (Yes)

---

c) Necessidade de rever na próxima visita?
c) Need to be reviewed during next visit?

◯ 0 = Não (No)
◯ 1 = Sim (Yes)

---

d) Outra patologia identificada?
d) Other pathology identified?

◯ 0 = Não (No)
◯ 1 = Sim (Yes)

Marque patologia no Checklist!

Check Pathology on Checklist!

---

2. Patologia 2:
2. Pathology 2:

---

a) Especifique:
a) Specify: __________________________________

---

b) Presente hoje?
b) Present today?

◯ 0 = Não (No)
◯ 1 = Sim (Yes)

---

c) Necessidade de rever na próxima visita?
c) Need to be reviewed during next visit?

◯ 0 = Não (No)
◯ 1 = Sim (Yes)

---

d) Outra patologia identificada?
d) Other pathology identified?

◯ 0 = Não (No)
◯ 1 = Sim (Yes)

Marque patologia no Checklist!

Check Pathology on Checklist!

3. Patologia 3:
3. Pathology 3:

a) Especifique:
a) Specify: __________________________________

b) Presente hoje?
b) Present today?
◯ 0 = Não (No)
◯ 1 = Sim (Yes)

c) Necessidade de rever na próxima visita?
c) Need to be reviewed during next visit?
◯ 0 = Não (No)
◯ 1 = Sim (Yes)

23. a) Exames solicitados durante esta visita?
23. a) Any investigations requested during this visit?
◯ 0 = Não (No)
◯ 1 = Sim (Yes)

b) Para cada um dos exames solicitados, forneça a informação solicitada:
b) For each investigation requested, please provide information:

Marque exame solicitado no Checklist!

Check Investigation on Checklist!

1. Exame 1:
1. Investigation 1:

a) Especifique:
a) Specify: __________________________________

b) Outro exame solicitado?
b) Another investigation requested?
◯ 0 = Não (No)
◯ 1 = Sim (Yes)

Marque exame solicitado no Checklist!

Check Investigation on Checklist!

2. Exame 2:
2. Investigation 2:

a) Especifique:
a) Specify: __________________________________

b) Outro exame solicitado?
b) Another investigation requested?
◯ 0 = Não (No)
◯ 1 = Sim (Yes)

Marque exame solicitado no Checklist!

Check Investigation on Checklist!

3. Exame 3:
3. Investigation 3:

a) Especifique:
a) Specify: __________________________________

24. a) Encaminhamento a especialista durante esta visita?
24. a) Any referral to a specialist during this visit?

◯ 0 = Não (No)
◯ 1 = Sim (Yes)

b) Para cada um dos encaminhamentos a especialistas, forneça a informação solicitada:
b) For each referral requested, please provide information:

Marque encaminhamento ao especialista no Checklist!

Check Referral on Checklist!

1. Encaminhamento - Especialista 1:
1. Referral 1:

a) Especifique:
a) Specify: __________________________________

b) Outro encaminhamento solicitado?
b) Another investigation requested?

◯ 0 = Não (No)
◯ 1 = Sim (Yes)

Marque encaminhamento ao especialista no Checklist!

Check Referral on Checklist!

2. Encaminhamento - Especialista 2:
2. Referral 2:

a) Especifique:
a) Specify: __________________________________

Observações:
Remarks:
