# Supplementary material for: Cohort profile: Study on Zika virus infection in Brazil (ZIKABRA study)
Source: PLoS One. 2021 Jan 5;16(1):e0244981. doi: 10.1371/journal.pone.0244981 (PMC7785242; doi:10.1371/journal.pone.0244981)
Supplement: S9 File — (PDF) [file pone.0244981.s009.pdf]

Confidential

SEG

Número de triagem: ______________________

A65921 - Persistência do vírus Zika nos fluidos corporais de pacientes com infecção pelo vírus Zika
Questionário de Seguimento

A65921 - Persistence of Zika virus in body fluids of patients with Zika virus infection
Follow-up Questionnaire

Centro:
Centre:

◯ 51 = Manaus - FMT
◯ 81 = Rio de Janeiro - FIOCRUZ
◯ 91 = Recife - HC

Número único de identificação:
Unique ID number: ______________________

Repetir Número único de identificação:
Repeat Unique ID number: ______________________

"Número de Identificação Única" e "Repetir Número de Identificação Única" estão diferentes, por favor verificar!

"Unique ID number" and "Repeat Unique ID number" are different, please verify!

Se Centro = 51 (Manaus - FMT), então "Número de Identificação Única" deve ser entre 151001 - 151300 ou 251001 - 251300!

If Centre = 51 (Manaus - FMT), then "Unique ID number" should be between 151001 - 151300 or 251001 - 251300!

Se Centro = 81 (Manaus - FMT), então "Número de Identificação Única" deve ser entre 181001 - 181300 ou 281001 - 281300!

If Centre = 81 (Manaus - FMT), then "Unique ID number" should be between 181001 - 181300 or 281001 - 281300!

Se Centro = 91 (Manaus - FMT), então "Número de Identificação Única" deve ser entre 191001 - 191300 ou 291001 - 291300!

If Centre = 91 (Manaus - FMT), then "Unique ID number" should be between 191001 - 191300 or 291001 - 291300!

INFORMAÇÃO SOBRE VISITA DE SEGUIMENTO
INFORMATION ON FOLLOW-UP VISIT

1. a) Data da visita:
1. a) Date of visit: ______________________

b) Profissional de saúde que preencheu o questionário (iniciais):
b) Health professional who completed the questionnaire (Initials):

◯ LHM = Luiz Maciel
◯ FAF = Francielen de Azevedo Furtado
◯ PCT = Pâmela
◯ NMR = Nágila Morais Rocha

b) Profissional de saúde que preencheu o questionário (iniciais):<br>b) Health professional who completed the questionnaire (Initials):	◯ FFS = Fernanda Figueiredo<br>◯ KEV = Kennya Valenca
2. a) Número da visita:<br>2. a) Visit number:	______________________
b) 1. Visita realizada:<br>b) 1. Visit completed:	◯ 1 = Realizada (Completed)<br>◯ 2 = Não realizada (Not completed)
2. Se Visita não realizada, por qual razão?<br>2. If Visit not completed, why?	◯ 1 = Não pode comparecer (Unable to come)<br>◯ 2 = Recusou a vir à clínica (Refused to come)<br>◯ 3 = Razão desconhecida (Reason unknown)<br>◯ 4 = Outro (Other)
Se Outro, especificar:<br>If Other, specify:	______________________
c) Tipo de visita:<br>c) Visit type:	◯ 1 = Programada (Scheduled)<br>◯ 2 = Não programada (Unscheduled)
d) Se Visita Não programada, razão:<br>d) If unscheduled visit, reason:	◯ 1 = Não foi possível fornecer uma ou mais amostras durante a última visita (Was unable to provide one or more sample during last visit)<br>◯ 2 = Sente-se doente (Feels sick)<br>◯ 3 = Estava doente durante a última visita e foi solicitado a retornar (Was sick during last visit and was asked to come back)<br>◯ 4 = Outro (Other)
Se Outro, especificar:<br>If Other, specify:	______________________
3. a) Depois de sua última visita de estudo, alguém em sua casa/parceiro sexual teve infecção pelo vírus Zika?<br>3. a) After your last study visit, has anyone in your home/any sexual partner got Zika virus infection?	◯ 0 = Não (No)<br>◯ 1 = Sim (Yes)<br>◯ 8 = Não sabe (Don't know)<br>◯ 9 = Recusou (Refused)
b) Se Sim, quem são essas pessoas?<br>b) If Yes, who are those persons?	

1. Pessoa 1:
1. Person 1:

- ◯ 1 = Marido/companheiro (Husband)
- ◯ 2 = Esposa/companheira (Wife)
- ◯ 3 = Parceria sexual (outro) (Sexual partner (other))
- ◯ 4 = Pai (Father)
- ◯ 5 = Mãe (Mother)
- ◯ 6 = Irmão (Brother)
- ◯ 7 = Irmã (Sister)
- ◯ 8 = Filho (Son)
- ◯ 9 = Filha (Daughter)
- ◯ 10 = Avô (Grand father)
- ◯ 11 = Avó (Grand mother)
- ◯ 12 = Outro (Other)

Se Outro, especificar:
If Other, specify: ______________________________

2. Pessoa 2:
2. Person 2:

- ◯ 1 = Marido/companheiro (Husband)
- ◯ 2 = Esposa/companheira (Wife)
- ◯ 3 = Parceria sexual (outro) (Sexual partner (other))
- ◯ 4 = Pai (Father)
- ◯ 5 = Mãe (Mother)
- ◯ 6 = Irmão (Brother)
- ◯ 7 = Irmã (Sister)
- ◯ 8 = Filho (Son)
- ◯ 9 = Filha (Daughter)
- ◯ 10 = Avô (Grand father)
- ◯ 11 = Avó (Grand mother)
- ◯ 12 = Outro (Other)

Se Outro, especificar:
If Other, specify: ______________________________

3. Pessoa 3:
3. Person 3:

- ◯ 1 = Marido/companheiro (Husband)
- ◯ 2 = Esposa/companheira (Wife)
- ◯ 3 = Parceria sexual (outro) (Sexual partner (other))
- ◯ 4 = Pai (Father)
- ◯ 5 = Mãe (Mother)
- ◯ 6 = Irmão (Brother)
- ◯ 7 = Irmã (Sister)
- ◯ 8 = Filho (Son)
- ◯ 9 = Filha (Daughter)
- ◯ 10 = Avô (Grand father)
- ◯ 11 = Avó (Grand mother)
- ◯ 12 = Outro (Other)

Se Outro, especificar:
If Other, specify: ______________________________

4. Pessoa 4:
4. Person 4:

◯ 1 = Marido/companheiro (Husband)
◯ 2 = Esposa/companheira (Wife)
◯ 3 = Parceria sexual (outro) (Sexual partner (other))
◯ 4 = Pai (Father)
◯ 5 = Mãe (Mother)
◯ 6 = Irmão (Brother)
◯ 7 = Irmã (Sister)
◯ 8 = Filho (Son)
◯ 9 = Filha (Daughter)
◯ 10 = Avô (Grand father)
◯ 11 = Avó (Grand mother)
◯ 12 = Outro (Other)

Se Outro, especificar:
If Other, specify: ________________________________

5. Pessoa 5:
5. Person 5:

◯ 1 = Marido/companheiro (Husband)
◯ 2 = Esposa/companheira (Wife)
◯ 3 = Parceria sexual (outro) (Sexual partner (other))
◯ 4 = Pai (Father)
◯ 5 = Mãe (Mother)
◯ 6 = Irmão (Brother)
◯ 7 = Irmã (Sister)
◯ 8 = Filho (Son)
◯ 9 = Filha (Daughter)
◯ 10 = Avô (Grand father)
◯ 11 = Avó (Grand mother)
◯ 12 = Outro (Other)

Se Outro, especificar:
If Other, specify: ________________________________

4. a) Depois de sua última visita de estudo, você foi hospitalizado(a)?
4. a) After your last study visit, have you been hospitalized?

◯ 0 = Não (No)
◯ 1 = Sim (Yes)

b) Que sintomas você teve?
b) What symptoms did you have? ________________________________

c) Que dia você foi ao hospital?
c) What day where you admitted in the hospital? ________________________________

d) Quantos dias você permaneceu no hospital?
d) How many days did you stay in the hospital? ________________________________

e) Qual foi o diagnóstico?
e) What was the diagnosis? ________________________________

f) Que tratamento você recebeu?
f) What treatment did you receive? ________________________________

5. Comparado à sua última visita, como esta a sua saúde geral e bem-estar?<br>5. Compared to your last visit, how is your overall health and well-being?	◯ 1 = O mesmo que eu senti na minha última visita de estudo (The same as how I felt at my last study visit)<br>◯ 2 = Pior do que na minha última visita de estudo (Worse than how I felt at my last study visit)<br>◯ 3 = Melhor do que na minha última visita de estudo (Better than how I felt at my last study visit)<br>◯ 4 = Não aplicável (Not applicable)<br>◯ 8 = Não sabe (Don't know)<br>◯ 9 = Recusou (Refused)
6. O(a) participante teve alguma reactivação/reinfecção ou complicação por Zika, outra patologia, exame ou encaminhamento a um especialista pendente durante a última visita?<br>6. Did the participant have any pending Zika reactivation/reinfection, other pathology, investigation or referal to a specialist during last visit?	◯ 0 = Não (No)<br>◯ 1 = Sim (Yes)
7. Presença de sintomas após a última visita:<br>7. Presence of symptoms since last visit:	
a) Sintomas de Zika:<br>a) Zika symptoms:	
1. Febre?<br>1. Fever?	◯ 0 = Não (No)<br>◯ 1 = Sim (Yes)
2. a) Erupção cutânea?<br>2. a) Skin rash?	◯ 0 = Não (No)<br>◯ 1 = Sim (Yes)
b) Erupção máculo-papular?<br>b) Maculopapular rash?	◯ 0 = Não (No)<br>◯ 1 = Sim (Yes)
3. Coceira na pele (prurido)?<br>3. Skin itching (pruritus)?	◯ 0 = Não (No)<br>◯ 1 = Sim (Yes)
4. Hiperemia conjuntival sem secreção e sem coceira?<br>4. Conjunctival hyperemia without secretions and without pruritus?	◯ 0 = Não (No)<br>◯ 1 = Sim (Yes)
5. Dor nas articulações (artralgia)?<br>5. Joint pain (arthralgia)?	◯ 0 = Não (No)<br>◯ 1 = Sim, apenas em uma articulação (Yes, only one joint)<br>◯ 2 = Sim, em duas ou mais articulações (Yes, in two joints or more)
6. Edema periarticular?<br>6. Periarticular edema?	◯ 0 = Não (No)<br>◯ 1 = Sim (Yes)

7. Sintoma(s) de Zika que necessitem consulta médica?<br>7. Presence of Zika symptom(s) that require to be seen by the doctor?	◯ 0 = Não (No)<br>◯ 1 = Sim (Yes)<br>(Até 3 semanas do recrutamento: responder Sim, apenas se houve agravamento dos sintomasApós 3 semanas do recrutamento: responder Sim (a não ser que o médico tenha indicado que não há necessidade de consulta) Till 3 weeks after recruitment: answer Yes only if this/these symptom(s) are worsening After 3 weeks after recruitment: answer Yes if any symptom present (except if the doctor said during a previous visit that this/these symptom(s) do not require to medical review))
b) Sintomas neurológicos:<br>b) Neurological symptoms:	
1. Sintomas motores (fraqueza muscular, perda de movimento, etc.)?<br>1. Motor symptoms (muscular weakness, loss of movement, etc.)?	◯ 0 = Não (No)<br>◯ 1 = Sim (Yes)
2. Sintomas sensitivos (dormência, queimação, formigamento, etc.)?<br>2. Sensitivity symptoms (sensation of numbness, burning, tingling, etc.)?	◯ 0 = Não (No)<br>◯ 1 = Sim (Yes)
3. Alteração do comportamento (irritabilidade, agitação, sonolência, etc.)?<br>3. Modification of behaviour (irritability, agitation, drowsiness, etc.)?	◯ 0 = Não (No)<br>◯ 1 = Sim (Yes)
4. Incoordenação motora (falta de precisão na coordenação dos movimentos, etc.)?<br>4. Lack of coordination in movements (lack of precision of coordination in movements, etc.)?	◯ 0 = Não (No)<br>◯ 1 = Sim (Yes)
5. Comprometimento dos nervos cranianos (visão, olfato, paladar, audição, movimento dos olhos, língua, face ou ombros, equilíbrio, diâmetro da pupila)?<br>5. Cranial nerves impairment (sight, smell, taste, earing, movement of the eyes, tongue, face or shoulders, balance, pupils diameter)?	◯ 0 = Não (No)<br>◯ 1 = Sim (Yes)
c) Outros sinais ou sintomas que requeiram atenção médica?<br>c) Other signs or symptoms requiring medical attention?	◯ 0 = Não (No)<br>◯ 1 = Sim (Yes)
Se Sim, especificar:<br>If Yes, specify:	__________________________
8. O(a) participante precisa ser visto pelo médico?<br>8. Does the participant need to be seen by the doctor?	◯ 0 = Não (No)<br>◯ 1 = Sim (Yes)

Se qualquer uma das perguntas 6, 7a7, 7b1 a 7b5 ou 7c for "Sim", então 8. deve ser "Sim"!

If any of the questions 6, 7a7, 7b1 to 7b5 or 7c is "Yes" then 8. should be "Yes"!

Se todas as perguntas 6, 7a7, 7b1 a 7b5 ou 7c forem "Não", então 8. deve ser "Não"!

If all questions 6, 7a7, 7b1 a 7b5 ou 7c are "No" then 8. should be "No"!

9. Peso:
9. Weight:

____
(Kg Formato XXX.XUtilizar ponto ao invés de vírgula para casas decimaisKg Format XXX.X Use period (full stop) instead of comma for decimal places)

☐ Não medido (Not measured)

10. Temperatura:
10. Temperature:

____
(°C Formato XX.XUtilizar ponto ao invés de vírgula para casas decimais°C Format XX.X Use period (full stop) instead of comma for decimal places)

☐ Não medida (Not measured)

11. Frequência cardíaca:
11. Heart rate:

____
(Batidas/minBeats/min)

☐ Não medida (Not measured)

12. Frequência respiratória:
12. Respiratory rate:

____
(Ciclos/minCycles/min)

☐ Não medida (Not measured)

13. Pressão arterial (sentado por pelo menos 5 minutos)
13. Blood pressure (sitting for at least 5 minutes)

a) Sistólica:
a) Systolic

____
(mmHg)

☐ Não medida (Not measured)

b) Diastólica:
b) Diastolic

____
(mmHg)

☐ Não medida (Not measured)

Observações:
Remarks:
