# Supplementary material for: Cohort profile: Study on Zika virus infection in Brazil (ZIKABRA study)
Source: PLoS One. 2021 Jan 5;16(1):e0244981. doi: 10.1371/journal.pone.0244981 (PMC7785242; doi:10.1371/journal.pone.0244981)
Supplement: S10 File — (PDF) [file pone.0244981.s010.pdf]

CLB

Número de triagem: ______________________

A65921 - Persistência do vírus Zika nos fluidos corporais de pacientes com infecção pelo vírus Zika
Questionário Clínico - B

A65921 - Persistence of Zika virus in body fluids of patients with Zika virus infection
Medical Questionnaire - B

Centro:
Centre:

- ◯ 51 = Manaus - FMT
- ◯ 81 = Rio de Janeiro - FIOCRUZ
- ◯ 91 = Recife - HC

Número único de identificação:
Unique ID number: ______________________

Repetir Número único de identificação:
Repeat Unique ID number: ______________________

"Número de Identificação Única" e "Repetir Número de Identificação Única" estão diferentes, por favor verificar!

"Unique ID number" and "Repeat Unique ID number" are different, please verify!

Se Centro = 51 (Manaus - FMT), então "Número de Identificação Única" deve ser entre 151001 - 151300 ou 251001 - 251300!

If Centre = 51 (Manaus - FMT), then "Unique ID number" should be between 151001 - 151300 or 251001 - 251300!

Se Centro = 81 (Manaus - FMT), então "Número de Identificação Única" deve ser entre 181001 - 181300 ou 281001 - 281300!

If Centre = 81 (Manaus - FMT), then "Unique ID number" should be between 181001 - 181300 or 281001 - 281300!

Se Centro = 91 (Manaus - FMT), então "Número de Identificação Única" deve ser entre 191001 - 191300 ou 291001 - 291300!

If Centre = 91 (Manaus - FMT), then "Unique ID number" should be between 191001 - 191300 or 291001 - 291300!

INFORMAÇÃO SOBRE VISITA DE SEGUIMENTO
INFORMATION ON FOLLOW-UP VISIT

1. a) Data da visita:
1. a) Date of visit: ______________________

2. a) Número da visita:
2. a) Visit number: ______________________

b) Tipo de visita:
b) Visit type:

- ◯ 1 = Programada (Scheduled)
- ◯ 2 = Não programada (Unscheduled)

Profissional de saúde que preencheu o questionário (iniciais):<br>Health professional who completed the questionnaire (Initials):	◯ CAB = Camila Botto<br>◯ ALA = Aline Alencar
Profissional de saúde que preencheu o questionário (iniciais):<br>Health professional who completed the questionnaire (Initials):	◯ CRB = Carlos Brito<br>◯ PAS = Paulo Sergio<br>◯ DAP = Danylo Pereira<br>◯ BRS = Braulio Silveira<br>◯ ALS = Aletheia Sampaio<br>◯ HIB = Hildenice Bernardes

PENDENTE DESDE A ÚLTIMA VISITA
PENDING SINCE LAST VISIT

3. a) Algum novo resultado de exames?<br>3. a) Any new result of investigations?	◯ 0 = Não (No)<br>◯ 1 = Sim (Yes)
b) Resultado do exame 1<br>b) Result of investigation 1	
1. Especifique a investigação:<br>1. Specify the investigation:	__________________________________
2. Resultado:<br>2. Result:	__________________________________
3. Outro novo resultado disponível de exames?<br>3. Other available result of investication?	◯ 0 = Não (No)<br>◯ 1 = Sim (Yes)
c) Resultado do exame 2<br>c) Result of investigation 2	
1. Especifique a investigação:<br>1. Specify the investigation:	__________________________________
2. Resultado:<br>2. Result:	__________________________________
3. Outro novo resultado disponível de exames?<br>3. Other available result of investication?	◯ 0 = Não (No)<br>◯ 1 = Sim (Yes)
d) Resultado do exame 3<br>d) Result of investigation 2	
1. Especifique a investigação:<br>1. Specify the investigation:	__________________________________
2. Resultado:<br>2. Result:	__________________________________
4. a) Algum novo resultado de encaminhamento a um especialista?<br>4. a) Any new result of referal to a specialist?	◯ 0 = Não (No)<br>◯ 1 = Sim (Yes)
b) Resultado de encaminhamento ao especialista 1<br>b) Result of referal to specialist 1	

1. Especifique o especialista:<br>1. Specify the secialist:	__________________________________
2. Resultado:<br>2. Result:	__________________________________
3. Outro novo resultado disponível de encaminhamento a um especialista?<br>3. Other available result of referal to a specialist?	◯ 0 = Não (No)<br>◯ 1 = Sim (Yes)
c) Resultado de encaminhamento ao especialista 2<br>c) Result of referal to specialist 2	
1. Especifique o especialista:<br>1. Specify the secialist:	__________________________________
2. Resultado:<br>2. Result:	__________________________________

SINTOMAS
SYMPTOMS

5. a) O(A) enfermeiro(a) identificou sintomas de Zika presente após a última visita?<br>5. a) Did the nurse identify the presence of Zika symptoms since last visit?	◯ 0 = Não (No)<br>◯ 1 = Sim (Yes)
b) Sintomas de Zika presentes após a última visita:<br>b) Zika symptoms present since last visit:	☐ 1 = Erupção máculo-papular que começou 48 horas após os primeiros sintomas (Maculo-papular rash that started within 48 hours after the first symptoms)<br>☐ 2 = Febre (Fever)<br>☐ 3 = Coceira na pele (prurido) (Skin itching (pruritus))<br>☐ 4 = Hiperemia conjuntival sem secreção e sem prurido (Conjunctival hyperemia without secretions and without pruritus)<br>☐ 5 = Dor nas articulações (artralgia) (Joint pain)<br>☐ 6 = Edema periarticular (Periarticular edema)
c) Esses sintomas são parte da fase inicial da doença de Zika ou podem ser sinais re-infecção/reativação de Zika?<br>c) Are those symptoms part of the inital phase of Zika symptoms or could they be signs of Zika reinfection/reactivation?	◯ 1 = Fase inicial da doença de Zika (Initial phase of Zika infection)<br>◯ 2 = Podem ser sinais de re-infecção/reativação de Zika (Could be signs of Zika reinfection/reactivation)<br>◯ 3 = Desconhecido (Unknown)
6. a) O(A) enfermeiro(a) identificou sintomas neurólogicos presentes após a última visita?<br>6. a) Did the nurse identify the presence of neurologocal symptoms since last visit?	◯ 0 = Não (No)<br>◯ 1 = Sim (Yes)

b) Sintomas neurológicos que ocorreram após a última visita:
b) Neurological symptoms present since last visit:

☐ 1 = Sintomas motores (fraqueza muscular, perda de movimento, etc.) (Motor symptoms)
☐ 2 = Sintomas sensitivos (dormência, queimação, formigamento, etc.) (Sensitivity symptoms)
☐ 3 = Alteração do comportamento (irritabilidade, agitação, sonolência, etc.) (Modification of beahaviour)
☐ 4 = Incoordenação motora (falta de precisão de coordenação em movimentos, etc.) (Lack of coordination in movements)
☐ 5 = Comprometimento dos nervos cranianos (visão, olfato, paladar, audição, movimentos dos olhos, língua, face ou ombros, equilíbrio, diâmetro da pupila, etc.) (Cranial nerves impairment)

---

c) Para cada um dos sintomas/sinais selecionados, forneça a informação solicitada:
c) For each of the selected signs/simptomas, provide the requested details:

---

1. Sintomas motores
1. Motor symptoms

---

a) Especifique:
a) Specify:

____

---

b) Presente após a última visita?
b) Present after last visit?

◯ 0 = Não (No)
◯ 1 = Sim (Yes)

---

c) Presente hoje?
c) Present today?

◯ 0 = Não (No)
◯ 1 = Sim (Yes)

---

2. Sintomas sensitivos
2. Sensitivity symptoms

---

a) Especifique:
a) Specify:

____

---

b) Presente após a última visita?
b) Present after last visit?

◯ 0 = Não (No)
◯ 1 = Sim (Yes)

---

c) Presente hoje?
c) Present today?

◯ 0 = Não (No)
◯ 1 = Sim (Yes)

---

3. Alteração do comportamento
3. Modification of beahaviour

---

a) Especifique:
a) Specify:

____

---

b) Presente após a última visita?
b) Present after last visit?

◯ 0 = Não (No)
◯ 1 = Sim (Yes)

---

c) Presente hoje?
c) Present today?

◯ 0 = Não (No)
◯ 1 = Sim (Yes)

---

4. Incoordenação motora
4. Lack of coordination in movements

a) Especifique:<br>a) Specify:	__________________________
b) Presente após a última visita?<br>b) Present after last visit?	◯ 0 = Não (No)<br>◯ 1 = Sim (Yes)
c) Presente hoje?<br>c) Present today?	◯ 0 = Não (No)<br>◯ 1 = Sim (Yes)
5. Comprometimento dos nervos cranianos<br>5. Cranial nerves impairment	
a) Especifique:<br>a) Specify:	__________________________
b) Presente após a última visita?<br>b) Present after last visit?	◯ 0 = Não (No)<br>◯ 1 = Sim (Yes)
c) Presente hoje?<br>c) Present today?	◯ 0 = Não (No)<br>◯ 1 = Sim (Yes)
7. a) Outros sintomas que ocorreram após a última visita?<br>7. a) Other symptoms that occurred after last visit?	◯ 0 = Não (No)<br>◯ 1 = Sim (Yes)
b) Para cada um dos sintomas identificados, forneça a informação solicitada:<br>b) For each of the symptoms identified, provided the requested information:	
1. Sintoma 1<br>1. Symptom 1	
a) Especifique:<br>a) Specify:	__________________________
b) Presente após a última visita?<br>b) Present after last visit?	◯ 0 = Não (No)<br>◯ 1 = Sim (Yes)
c) Presente hoje?<br>c) Present today?	◯ 0 = Não (No)<br>◯ 1 = Sim (Yes)
d) Outro sintoma identificado?<br>d) Other symptom identified?	◯ 0 = Não (No)<br>◯ 1 = Sim (Yes)
2. Sintoma 2<br>2. Symptom 2	
a) Especifique:<br>a) Specify:	__________________________
b) Presente após a última visita?<br>b) Present after last visit?	◯ 0 = Não (No)<br>◯ 1 = Sim (Yes)
c) Presente hoje?<br>c) Present today?	◯ 0 = Não (No)<br>◯ 1 = Sim (Yes)

d) Outro sintoma identificado?<br>d) Other symptom identified?	◯ 0 = Não (No)<br>◯ 1 = Sim (Yes)
3. Sintoma 3<br>3. Symptom 3	
a) Especifique:<br>a) Specify:	__________________________________
b) Presente após a última visita?<br>b) Present after last visit?	◯ 0 = Não (No)<br>◯ 1 = Sim (Yes)
c) Presente hoje?<br>c) Present today?	◯ 0 = Não (No)<br>◯ 1 = Sim (Yes)
d) Outro sintoma identificado?<br>d) Other symptom identified?	◯ 0 = Não (No)<br>◯ 1 = Sim (Yes)
4. Sintoma 4<br>4. Symptom 4	
a) Especifique:<br>a) Specify:	__________________________________
b) Presente após a última visita?<br>b) Present after last visit?	◯ 0 = Não (No)<br>◯ 1 = Sim (Yes)
c) Presente hoje?<br>c) Present today?	◯ 0 = Não (No)<br>◯ 1 = Sim (Yes)
d) Outro sintoma identificado?<br>d) Other symptom identified?	◯ 0 = Não (No)<br>◯ 1 = Sim (Yes)
5. Sintoma 5<br>5. Symptom 5	
a) Especifique:<br>a) Specify:	__________________________________
b) Presente após a última visita?<br>b) Present after last visit?	◯ 0 = Não (No)<br>◯ 1 = Sim (Yes)
c) Presente hoje?<br>c) Present today?	◯ 0 = Não (No)<br>◯ 1 = Sim (Yes)

EXAME FÍSICO
EXAMINATION

Informe os achados presentes hoje
Report the signs present today

8. Gerais:<br>8. General signs:	◯ 0 = Não (No)<br>◯ 1 = Sim (Yes)
Se Sim, especificar:<br>If Yes, specify:	__________________________________
9. Hemorrágicos:<br>9. Bleeding:	◯ 0 = Não (No)<br>◯ 1 = Sim (Yes)
Se Sim, especificar:<br>If Yes, specify:	__________________________________
10. Cutâneo ou da mucosa/garganta:<br>10. Skin and mucosa/throat signs:	◯ 0 = Não (No)<br>◯ 1 = Sim (Yes)
Se Sim, especificar:<br>If Yes, specify:	__________________________________
11. Sistema circulatório/respiratório:<br>11. Respiratory/cardiac abnormalities:	◯ 0 = Não (No)<br>◯ 1 = Sim (Yes)
Se Sim, especificar:<br>If Yes, specify:	__________________________________
12. Abdominais:<br>12. Abdominal abnormalities:	◯ 0 = Não (No)<br>◯ 1 = Sim (Yes)
Se Sim, especificar:<br>If Yes, specify:	__________________________________
13. Reumatológicos:<br>13. Joints abnormalities:	◯ 0 = Não (No)<br>◯ 1 = Sim (Yes)
Se Sim, especificar:<br>If Yes, specify:	__________________________________
14. Neurológicos:<br>14. Neurological abnormalities:	◯ 0 = Não (No)<br>◯ 1 = Sim (Yes)
Se Sim, especificar:<br>If Yes, specify:	__________________________________
15. Cadeia ganglionar:<br>15. Lymphe nodes:	◯ 0 = Não (No)<br>◯ 1 = Sim (Yes)
Se Sim, especificar:<br>If Yes, specify:	__________________________________
16. Outra alterações:<br>16. Other abnormalities:	◯ 0 = Não (No)<br>◯ 1 = Sim (Yes)

Se Sim, especificar:
If Yes, specify: ______________________

DIAGNÓSTICO
DIAGNOSIS

17. a) Alguma patologia ocorreu após a última visita?
17. a) Any pathology during this visit?
◯ 0 = Não (No)
◯ 1 = Sim (Yes)

b) Para cada um das patologias identificadas, forneça a informação solicitada:
b) For each pathology present during this period, please provide the information requested:

1. Patologia 1
1. Pathology 1

a) Especifique:
a) Specify: ______________________

b) Presente hoje?
b) Present today?
◯ 0 = Não (No)
◯ 1 = Sim (Yes)

c) Necessidade de rever na próxima visita?
c) Need to be reviewed during next visit?
◯ 0 = Não (No)
◯ 1 = Sim (Yes)

d) Outra patologia identificada?
d) Other pathology identified?
◯ 0 = Não (No)
◯ 1 = Sim (Yes)

2. Patologia 2
2. Pathology 2

a) Especifique:
a) Specify: ______________________

b) Presente hoje?
b) Present today?
◯ 0 = Não (No)
◯ 1 = Sim (Yes)

c) Necessidade de rever na próxima visita?
c) Need to be reviewed during next visit?
◯ 0 = Não (No)
◯ 1 = Sim (Yes)

d) Outra patologia identificada?
d) Other pathology identified?
◯ 0 = Não (No)
◯ 1 = Sim (Yes)

3. Patologia 3
3. Pathology 3

a) Especifique:
a) Specify: ______________________

b) Presente hoje?
b) Present today?
◯ 0 = Não (No)
◯ 1 = Sim (Yes)

c) Necessidade de rever na próxima visita?
c) Need to be reviewed during next visit?
◯ 0 = Não (No)
◯ 1 = Sim (Yes)

18. a) Exames solicitados durante esta visita?<br>18. a) Any investigations requested during this visit?	◯ 0 = Não (No)<br>◯ 1 = Sim (Yes)
b) Para cada um dos exames solicitados, forneça a informação solicitada:<br>b) For each investigation requested, please provide information:	
1. Exame 1<br>1. Investigation 1	
a) Especifique:<br>a) Specify:	__________________________________
b) Outro exame solicitado?<br>b) Another investigation requested?	◯ 0 = Não (No)<br>◯ 1 = Sim (Yes)
2. Exame 2<br>2. Investigation 2	
a) Especifique:<br>a) Specify:	__________________________________
b) Outro exame solicitado?<br>b) Another investigation requested?	◯ 0 = Não (No)<br>◯ 1 = Sim (Yes)
3. Exame 3<br>3. Investigation 3	
a) Especifique:<br>a) Specify:	__________________________________
19. a) Encaminhamento a especialista durante esta visita?<br>19. a) Any referral to a specialist during this visit?	◯ 0 = Não (No)<br>◯ 1 = Sim (Yes)
b) Para cada um dos encaminhamentos a especialistas, forneça a informação solicitada:<br>b) For each referral requested, please provide information:	
1. Encaminhamento - Especialista 1<br>1. Referral 1	
a) Especifique:<br>a) Specify:	__________________________________
b) Outro encaminhamento solicitado?<br>b) Another referral requested?	◯ 0 = Não (No)<br>◯ 1 = Sim (Yes)
2. Encaminhamento - Especialista 2<br>2. Referral 2	
a) Especifique:<br>a) Specify:	__________________________________

Observações:
Remarks:
