# Supplementary material for: Cohort profile: Study on Zika virus infection in Brazil (ZIKABRA study)
Source: PLoS One. 2021 Jan 5;16(1):e0244981. doi: 10.1371/journal.pone.0244981 (PMC7785242; doi:10.1371/journal.pone.0244981)
Supplement: S11 File — (PDF) [file pone.0244981.s011.pdf]

FIM

Número de triagem: __________________________________

A65921 - Persistência do vírus Zika nos fluidos corporais de pacientes com infecção pelo vírus Zika
Questionário de fim da participação no estudo

A65921 - Persistence of Zika virus in body fluids of patients with Zika virus infection
End of participation in the study questionnaire

Centro:
Centre:

- ◯ 51 = Manaus - FMT
- ◯ 81 = Rio de Janeiro - FIOCRUZ
- ◯ 91 = Recife - HC

Número único de identificação:
Unique ID number: __________________________________

Repetir Número único de identificação:
Repeat Unique ID number: __________________________________

"Número único de identificação" e "Repetir Número único de identificação" estão diferentes, por favor verificar!

"Unique ID number" and "Repeat Unique ID number" are different, please verify!

Se Centro = 51 (Manaus - FMT), então "Número de Identificação Única" deve ser entre 151001 - 151300 ou 251001 - 251300!

If Centre = 51 (Manaus - FMT), then "Unique ID number" should be between 151001 - 151300 or 251001 - 251300!

Se Centro = 81 (Manaus - FMT), então "Número de Identificação Única" deve ser entre 181001 - 181300 ou 281001 - 281300!

If Centre = 81 (Manaus - FMT), then "Unique ID number" should be between 181001 - 181300 or 281001 - 281300!

Se Centro = 91 (Manaus - FMT), então "Número de Identificação Única" deve ser entre 191001 - 191300 ou 291001 - 291300!

If Centre = 91 (Manaus - FMT), then "Unique ID number" should be between 191001 - 191300 or 291001 - 291300!

VISITA
VISIT

1. Data da conclusão do questionário:
1. Date of questionnaire completion: __________________________________

2. Profissional de saúde que preencheu o questionário (iniciais):
2. Health professional who completed the questionnaire (Initials):

- ◯ LHM = Luiz Maciel
- ◯ FAF = Francielen de Azevedo Furtado
- ◯ PCT = Pâmela
- ◯ NMR = Nágila Morais Rocha

2. Profissional de saúde que preencheu o questionário (iniciais):<br>2. Health professional who completed the questionnaire (Initials):	◯ FFS = Fernanda Figueiredo<br>◯ KEV = Kennya Valenca
3. a) Status no final da participação no estudo:<br>3. a) Status at the end of participation in the study:	◯ 1 = Dispensado (seguimento completo) 1 = Discharged (completed follow-up)<br>◯ 2 = Mudou-se para uma área tornando o seguimento impossível 2 = Moved to an area making follow-up impossible<br>◯ 3 = Recusou-se a continuar no estudo 3 = Refused to continue in the study<br>◯ 4 = Perda de seguimento 4 = Lost to follow-up<br>◯ 5 = Engravidou e foi descontinuada 5 = Became pregnant and was discontinued<br>◯ 6 = Homem incapaz de fornecer amostra de sêmen por duas visitas consecutivas 6 = Man unable to provide a semen sample at two consecutive visits<br>◯ 7 = Falecido 7 = Deceased<br>◯ 9 = Outro 9 = Other
Se Outro, especificar:<br>If Other, specify:	__________________________________
b) Data do final da participação no estudo:<br>b) Date of end of participation in the study:	__________________________________

Se 1 = Dispensado, marcar a data da última visita de estudo (V17)

If 1 = Discharged, report date of last study visit (V17)

Se 2 = Mudou-se para uma área tornando seguimento impossível, marcar a data da última comunicação com a equipe do estudo, ou a data da última vez que foi visto se não puder ser contactado

If 2 = Moved to an area making follow-up impossible, report date of last communication with study staff or date last seen if cannot be contacted

Se 3 = Recusou-se a continuar no estudo, marcar a data da última comunicação com a equipe do estudo

If 3 = Refused to continue in the study, report date of last communication with study staff

Se 4 = Perda de seguimento, marcar a data da última comunicação com a equipe do estudo ou a data da última visita

If 4 = Lost to follow-up, report date of last communication/contact or last visit date

Se 5 = Engravidou e foi descontinuada, marca a data em que a participante foi informada da interrupção de participação no estudo devido à gravidez

If 5 = Became pregnant and was discontinued, report date when participant was informed of study discontinuation due to pregnancy

Se 6 = Homem incapaz de fornecer amostra de sêmen em duas visitas consecutivas, marcar a data em que o participante foi informado da interrupção de particpação no estudo devido a incapacidade de fornecer esta amostra

If 6 = Man unable to provide a semen sample at two consecutive visits, report date when participant was informed of study discontinuation due to failure to provide semen sample

Se 7 = Falecido, marcar a data de falecimento

If 7 = Deceased, report date of death

Se 9 = Outro, marcar a data da última comunicação com a equipe do estudo ou a data da última visita

If 9 = Other, report date of last communication/contact or last visit date

---

c) Se 3 = Recusou-se a continuar no estudo, qual é a razão?
c) If 3 = Refused to continue in the study, what is the reason?

- ○ 1 = Razão pessoal 1 = Personal reason
- ○ 2 = Problema de saude 2 = Health problem
- ○ 3 = Outro 3 = Other

Se Outro, especificar:
If Other, specify: ________________________________

---

d) Se 5 = Engravidou e foi descontinuada, marcar a data em que a gravidez começou (com base, por exemplo, na data do primeiro dia do último período menstrual, resultado de um ultra-som, etc.).
d) If 5 = Became pregnant and was discontinued, report date pregnancy started (based for example on the date of the first day of the last menstruation period, the result of an ultrasound, etc.).

Dia:
Day:

- ○ Desconhecido
- ○ 1
- ○ 2
- ○ 3
- ○ 4
- ○ 5
- ○ 6
- ○ 7
- ○ 8
- ○ 9
- ○ 10
- ○ 11
- ○ 12
- ○ 13
- ○ 14
- ○ 15
- ○ 16
- ○ 17
- ○ 18
- ○ 19
- ○ 20
- ○ 21
- ○ 22
- ○ 23
- ○ 24
- ○ 25
- ○ 26
- ○ 27
- ○ 28
- ○ 29
- ○ 30
- ○ 31

Mês:<br>Month:	◯ Desconhecido<br>◯ Jan<br>◯ Fev<br>◯ Mar<br>◯ Abr<br>◯ Mai<br>◯ Jun<br>◯ Jul<br>◯ Ago<br>◯ Set<br>◯ Out<br>◯ Nov<br>◯ Dez

Ano:
Year:

◯ Desconhecido
◯ 1970
◯ 1971
◯ 1972
◯ 1973
◯ 1974
◯ 1975
◯ 1976
◯ 1977
◯ 1978
◯ 1979
◯ 1980
◯ 1981
◯ 1982
◯ 1983
◯ 1984
◯ 1985
◯ 1986
◯ 1987
◯ 1988
◯ 1989
◯ 1990
◯ 1991
◯ 1992
◯ 1993
◯ 1994
◯ 1995
◯ 1996
◯ 1997
◯ 1998
◯ 1999
◯ 2000
◯ 2001
◯ 2002
◯ 2003
◯ 2004
◯ 2005
◯ 2006
◯ 2007
◯ 2008
◯ 2009
◯ 2010
◯ 2011
◯ 2012
◯ 2013
◯ 2014
◯ 2015
◯ 2016
◯ 2017
◯ 2018
◯ 2019

e) Se 7 = Falecido, descrever a principal causa do falecimento:
e) If 7 = Deceased, describe the main cause of death:

____

Observações:
Remarks:
