# Supplementary material for: Cohort profile: Study on Zika virus infection in Brazil (ZIKABRA study)
Source: PLoS One. 2021 Jan 5;16(1):e0244981. doi: 10.1371/journal.pone.0244981 (PMC7785242; doi:10.1371/journal.pone.0244981)
Supplement: Attachment — Submitted filename: Response to Reviewers_18.12.2020.docx [file pone.0244981.s013.docx]

Manuscript number: **PONE-D-20-28958**

Title: Cohort profile: Study on the persistence of Zika virus in body fluids of patients with ZIKV infection in Brazil (ZIKABRA Study)

PLOS ONE

**Corresponding author:** Guilherme Calvet

| REVIEWER COMMENTS | RESPONSE |
| --- | --- |
| REVIEWER 2 | |
| Authors answer or/and corrected minor suggestions. However, in the answer to comments, they mentioned that the purpose/objective of this paper was only to report the profile of a cohort and delete any information on viral persistence, submitting results and virologic data to a future publication dedicated to this purpose. They answered only to the minor comments, stayed evasive and avoided the question relative to presence of ZIKV keeping this point for a future publication. I therefore note that this paper is only about the cohort profile but not on ZIKV persistence in body fluid contrary to what the title suggests. | We agree with this pertinent comment.  We changed the manuscript title to: “Cohort profile: Study on Zika virus infection in Brazil (ZIKABRA Study)”  We also changed the short title and add a sentence in the abstract section to better reflect the objective of the manuscript. |
